# Close and more distant relatives are associated with child mortality risk in historical Finland

**DOI:** 10.1017/ehs.2024.47

**Published:** 2025-01-20

**Authors:** Mirkka Lahdenperä, Milla Salonen, Takayuki Hiraoka, Martin W. Seltmann, Jari Saramäki, Virpi Lummaa

**Affiliations:** 1 Department of Biology, University of Turku, Turku, Finland; 2 Department of Computer Science, Aalto University, Espoo, Finland

**Keywords:** Kin networks, social relationships, child survival, maternal and paternal lineages, Relatives may aid or hinder child survival, depending on wealth and lineage, offering insights into cooperative breeding

## Abstract

Humans are characterised as cooperative breeders, as not only the parents but also other members of the social group take part in raising offspring. The individuals who invest most in childrearing are usually the more closely related individuals. However, most studies have concentrated on close kin and the effects of more distant kin remain unknown. Here, we investigated the associations of child mortality (<5 years, *n* = 32,000 children) with the presence of 36 different types of relatives, divided by lineage and sex, in a historical Finnish population. We found that the presence and greater number of several paternal relatives were associated with an increase in child mortality and many of these associations were seen among the wealthiest families, due to inheritance practices and shared resources. The presence of the maternal grandmother was associated with a decrease in child mortality and the most among poorer families, who probably needed the grandmother’s contribution more than the wealthy. Our results bring new insights into the importance of kin and suggest that relatives can provide support or other resources but also compete for limited resources and care. The results give a broader perspective of human family life and increase understanding of the evolution of cooperative breeding.

## Graphical abstract

**Figure:**
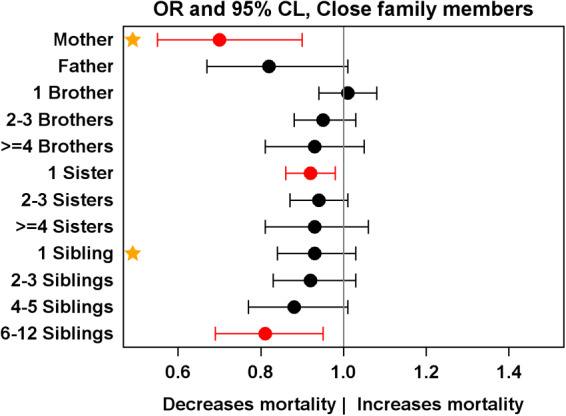

## 1. Introduction

Human life history is characterised by several unique features (Hawkes & Paine, 2006). Human infants are born helpless and dependent on parental care, not only for the first years of life, but for an extended period of time (Bogin & Varea, 2017). Still, compared to closely related species, humans have shorter inter-birth intervals, resulting in multiple different-aged dependants requiring high amounts of parental investments at the same time (Kramer & Otárola-Castillo, 2015; Robson et al., 2006). To meet the substantial costs of raising children, not only the parents but also other members of the social group take part in raising offspring. Thus, humans have been characterised as cooperative breeders (Hrdy, 2009; Mace & Sear, 2005).

Apart from parents, those who tend to invest significantly in childcare are usually closely related individuals. This is in line with Hamilton’s kin selection theory (Hamilton, 1964). The more closely the helpers are related to the children, the more they gain inclusive fitness benefits from helping. Investing in the well-being and survival of their genetic relatives indirectly promotes the transmission of their own genes to future generations, even if it comes at a cost to their own reproductive success. Additionally, closely related individuals are more likely to have shared interests and reciprocal relationships than more distantly related or unrelated individuals (Kramer, 2021). This can motivate them to invest time, resources, and effort in childcare. The help received from kin has been connected to beneficial effects on the physical and mental health of the children as well as higher child survival rates in pre-industrial as well as contemporary populations (Kramer, 2010; Sear & Coall, 2011; Tanskanen & Danielsbacka, 2018).

Particularly older generations are expected to invest in childcare, as transfers usually flow from older to younger generations (Lee, 2003, 2008). In line with this, post-reproductive grandmothers have been found to improve child survival widely. Especially, the presence of maternal grandmothers has been associated with higher child survival after weaning (Beise, 2005; Beise & Voland, 2002; Chapman et al., 2021; Engelhardt et al., 2019; Heath, 2003; Leonetti et al., 2005; Ragsdale, 2004; Sear et al., 2000, 2002; Sheppard & Sear, 2016; Tymicki, 2009; Voland & Beise, 2002). Another notable group of helpers, pre-reproductive siblings (‘helpers-at-the-nest’), have been found to increase child survival potentially through taking part in child rearing (Beise, 2005; Crognier et al., 2001, 2002; Nitsch et al., 2013; Sear, 2008; Sear et al., 2002). Also, other relatives, such as aunts and uncles, have been linked to higher child survival in some populations (Borgerhoff Mulder, 2007; Heath, 2003; Nitsch et al., 2014).

Even though kin can provide help, the relationships can also be harmful for child survival. Relationships with relatives may include competitive interactions, as the interests between the relatives may not be always compatible, resulting in decreased child survival. Accordingly, theory predicts that individuals may try to optimise their own survival, well-being and reproduction, even if it may be detrimental to kin (Hamilton, 1964). For example, in some patrilocal historical populations, the presence of paternal grandmothers has been found to have detrimental impacts on child survival and often the effects have been seen during infancy (Beise & Voland, 2002; Chapman et al., 2019; Jamison et al., 2002; Voland & Beise, 2002). However, in historical Germany and Poland, the presence of paternal grandmothers increased child survival during the first year of a child’s life (Kemkes-Grottenthaler, 2005; Tymicki, 2009). In addition, in a matrilineal society in Malawi, maternal grandmothers and maternal aunts increased child mortality, but these associations were modified by resource ownership: higher mortality was seen in households where women owned land and high mortality was suggested to emerge from resource competition between matrilineal kin (Sear, 2008). Therefore, besides genetic relatedness, the associations between relatives may depend on the cultural and socioeconomic contexts as well as child and relative characteristics (Chapman et al., 2018, 2019, 2021; Fox et al., 2010; Sear & Coall, 2011), influencing the direction of associations as well as their magnitude on child survival.

Although the associations between kin presence and child survival have been extensively investigated in pre-industrial and contemporary populations, there are some important limitations. The studies thus far have been confined to the most closely related kin (siblings, parents, grandparents, aunts, and uncles). Few studies have investigated several types of kin at the same time, instead concentrating on a specific relative type, such as grandmothers, as their helping behaviour has been linked to the evolution of post-reproductive lifespan in women (e.g. Chapman et al., 2019; Hawkes et al., 1998; Sear & Coall, 2011; Voland et al., 2005). Moreover, many studies have investigated either the maternal or paternal lineage, although both lineages warrant investigation simultaneously. Because individuals commonly maintain lifelong relationships with their natal families, despite marriage, emigration, and establishing families of their own, the kin provides foundations for regular social interaction, potentially influencing child survival. Importantly, there is a need for a more extensive understanding of broader networks involving relatives, especially within the same study setting, enabling meaningful comparisons between family members.

In this study, we investigated the associations of child mortality during the first five years of life with the presence (i.e. being alive) of 36 different types of relatives, divided by lineage and sex, in a historical Finnish population. We utilised an extensive family pedigree dataset that includes 31,392 children born between 1732 and 1879 with their family network known at the time of birth and thereafter. We considered only relatives whose degree of relatedness to the focal child was at least 0.125, and the kin network expanded horizontally to include first-cousins and vertically to great-grandparents. We used discrete-time event models that account for the yearly variation in the number of each relative type alive on the mortality of the children. We also investigate if child age and family socioeconomic status modify the associations between the presence of relatives and child mortality (interactions of age and family socioeconomic status (SES) with the presence of relative). In this pre-industrial Finnish population, child mortality and fertility were high (Chapman et al., 2021; Lahdenperä et al., 2011; Turpeinen, 1973). Residence was typically patrilocal, and the inheritance system favoured the eldest son, but the parents lived usually nearby all their children (Moring, 1998). Thus, both close as well as more distant kin were likely to live relatively close to each other. Previous studies in this population have investigated the importance of close kin (the relatedness varying between 0.25 and 0.50) such as parents (Lahdenperä et al., 2011), siblings (Nitsch et al., 2013), grandmothers (Chapman et al., 2019, 2021; Lahdenperä et al., 2004; Ukonaho et al., 2023), grandfathers (Lahdenperä et al., 2007), and aunts and uncles (Nitsch et al., 2014) on child survival. However, the associations with more distant relatives and their lineage and sex-dependent differences are unknown as well as the relative importance of all relatives.

We predict that (i) the more closely related kin will reduce child mortality more than more distantly related kin in line with kin selection theory (Hamilton, 1964); (ii) maternal relatives decrease child mortality more than paternal relatives due to more intense resource competition with paternal kin (Chapman et al., 2019; Lahdenperä et al., 2012; Nitsch et al., 2014; Pettay et al., 2016) and the cross-cultural matrilateral bias in alloparenting, suggested to result from paternity uncertainty in paternal kin and mother’s preferences to invest in maternal kin (Perry & Daly, 2017); (iii) female relatives decrease child mortality more than male relatives due to division of labour in historical Finland (women were more involved in childcare than men), empirical evidence showing females usually increase child survival more than males (e.g. Sear & Coall, 2011; Sear & Mace, 2008) and sex-specific reproductive strategies (females usually invest more in offspring than males, as reproduction is more costly for females and males invest more in mating competition) (Euler, 2011; Trivers, 1972); (iv) the relatives from older generations decrease child mortality more than relatives from the same generation due to intergenerational transfer theory (Lee, 2003, 2008) and potentially competitive interactions within the same generation (Nitsch et al., 2013). Finally, we predict that (v) the age of the focal child and family SES may modulate the associations between the presence of the relative and child mortality as younger children and families with poorer resources are likely to need help most but might also be most vulnerable to negative effects from competition (Chapman et al., 2018, 2021). Clarifying how the presence of relatives is associated with child mortality in these contexts can offer a broader perspective of human family life and also give important insights into the evolution of family, cooperative breeding and conflicting interactions in humans.

## 2. Methods

### 2.1 Study population

We utilised large demographic data collected from historical church records kept by the Lutheran church in Finland, enabling us to investigate how the presence of various relatives is associated with early childhood survival in a pre-industrial population. The Lutheran church records of Sweden–Finland are among the oldest population records in Europe dating back to the early eighteenth century (Luther & Erjos, 1993). The demographic data have been compiled from various historical church registers, including all births, deaths, marriages, and inter-parish movements in the country.

Pre-industrial Finland was a poor rural agrarian society (Soininen, 1974), where western Finland was characterised by permanent open-field agriculture, whereas slash-and-burn cultivation prevailed in the east. The climatic and living conditions were harsh (Holopainen & Helama, 2009). The main staples were rye and barley, but famines were common due to failures in crops (Voutilainen, 2016). The healthcare system was undeveloped and at the end of the nineteenth century the lack of physicians was still significant (Saarivirta et al., 2012). Infectious diseases were among the most common causes of death in Finland (Saarivirta et al., 2012; Ukonaho et al., 2022, 2023). The mating system was monogamous, and both divorce and extra-marital affairs were forbidden (Sundin, 1992). Family was one of the most important social institutions of the time, and its functions were numerous, bringing social and economic security as well as connecting kin networks. Residence was patrilocal and in western Finland, co-residence between (grand)parents and one married child was practiced by most farmers, whereas in eastern Finland, multiple joint family households with horizontal extension (usually brothers’ families) were also frequently found (Moring, 1998; Pettay et al., 2016, 2018). In east, the wealth of the household was dependent on the number of adult men in the unit as a larger area could be cultivated for slash-and-burn agriculture (Moring, 1998). Therefore, the large number of extended and multiple family households was the result of inheritance practices and economic systems. In general, transfer of land on the male line favoured the eldest son, but in the east, a brother could also inherit the land. The law stipulated equal inheritance between siblings, but daughters received half of a son’s share (e.g. cattle, money, bedding, textiles) (Moring, 1998). Men got an equal share of crop, money, and tools whereas women were excluded from ownership connected with agriculture (Moring, 1998). The one who got the farm was also responsible for the upkeep of his younger siblings until they reached the age to marry (Moring, 1998). The situation of the landless was very different from that of the farming group as they had little to give to their children. Landless households were smaller and less likely to have intergenerational co-residence. The number of children born and household sizes were greater among landowners than landless (Moring, 1998; Pettay et al., 2007). Migration rates were low and the migration distance from the natal parish was short, with particularly females often dispersing to the birthplace of their spouse (Nitsch et al., 2016). Therefore, kin, particularly patrilineal kin, were likely to live nearby, due to patrilocal residence patterns (Moring, 1998). The transformation from an agrarian to an industrial country and the demographic transition started relatively late in Finland, in the 1880s (Singleton, 1998), with gradual improvements in healthcare, income, birth control methods, transport options, and overall living standards.

The material used in this study has been gathered by following the same family lines through different times, collecting information on all relatives born to all individuals, regardless of their lifespan (exact or censored) or moving to other parishes in Finland or abroad. Thus, this study includes all children in the data set born between 1732 and 1879 with information on their death date (or censoring), mother’s identity and her presence, the presence of at least one relative, and adjusted covariates, which results in 31,392 children with 137,007 observations from birth to age 5 (Table 1). The children were born in several parishes around Finland and were categorised into four larger regions: Central and Eastern Finland, Karelia and Ingria, Ostrobothnia (including Lapland), and Southwest and Southern Finland. The father’s occupation was used to stratify children and their families into three SES categories: high (e.g. landowners, clergy, and merchants), moderate (e.g. tenant farmers, craftsmen, and fishermen), and low (e.g. farmless families and servants) (Pettay et al., 2007).

**Table 1.** Characteristics of the children and their survival to age 5 in the study population in historical Finland. The sample includes all children who had information on their survival to age 5 (or censoring before age 5) and all adjusted covariates in the survival models (fixed terms: child age (and squared age), twin status, sex, birth year, birth order, mother’s age (and squared mother’s age), living region, family SES, repeated term: mother’s id). *P*-values indicate the significance of the variable on child survival to 5 years of age (discrete time event model, *N* = 31,392 children)

Characteristics	*N* (%)/mean (SD)	Survived to age 5, *N* (%)	*P*-value
All	31,392	23,020 (73.33)	<0.0001^1^
Mothers	6,994		
Censored before age 5	190 (0.6%)		
Sex			0.0004
Boy	15,957 (50.83)	11,581 (72.57)	
Girl	15,435 (49.17)	11,439 (74.11)	
Twin			<0.0001
No	30,411 (96.88)	22,504 (74.00)	
Yes	981 (3.13)	516 (52.60)	
Birth year (1732−1879)	1,841.81 (29.94)		0.67
Birth order (1 − 18)			<0.0001
1	6,404 (20.40)	4,828 (75.39)	
2 − 4	14,612 (46.55)	10,892 (74.54)	
≥ 5	10,376 (33.05)	7,300 (70.35)	
Mother’s age at birth (15−50 years)	31.15 (6.38)		0.02^2^
Family SES			<0.0001
High	15,300 (48.74)	11,100 (72.54)	
Moderate	11,243 (35.81)	8,506 (75.66)	
Low	4,849 (15.45)	3,414 (70.40)	
Region			<0.0001
Central Finland	11,154 (35.53)	8,317 (74.57)	
Eastern Finland	2,338 (8.08)	1,674 (71.60)	
Northern Finland	9,180 (29.24)	6,563 (71.50)	
Southwest Finland	8,520 (27.14)	6,466 (75.89)	

1 Child age: β = −0.83, quadratic child age β = 0.10, *P-*value < 0.0001.

2 Mother’s age: β = −0.04; quadratic mother’s age β = 0.0007, *P*-value = 0.01.

### 2.2 Statistical analyses

All analyses were conducted using the SAS 9.4 statistical package (SAS Institute Inc., Cary, North Carolina). The level of significance was set at *p*-value < 0.05.

#### 2.3.1 Child mortality and presence of relatives

In the analyses, we focused on child mortality during the first five years of life, as the mortality rate was typically high in pre-industrial Finland in childhood (31%) and 80% of these deaths happened before the age of 5 (Chapman et al., 2021). Survival of each focal child was coded as a binomial time-varying variable, dead (0) or alive (1), at each age from birth to the age of 5 years, based on their exact death date. Children without a recorded date of death were censored at the last age they were known to be alive. We excluded children who died within a week of the death of their mother before the age of 5 to remove coincidental deaths due to external factors such as shared diseases (*n* = 161). We also excluded children who died within a week from birth as these deaths are likely to result from preterm births, intrapartum-related complications or birth defects (*n* = 1979). In both of these cases, it is unlikely that the presence of relatives could have a significant association with the survival of the children. By reconstructing the genealogy of the studied population, identifying the relatives of each child, and calculating the overlap in lifespan between the child and each relative, we determined the presence and number of 36 different types of relatives (including mother, father, siblings, (great) aunts and uncles, (great) grandparents, and cousins), classified by the lineage (maternal vs. paternal) and sex, for each focal child for each year from birth to the age of 5 years. The limit for relatedness value was set at 0.125. The presence of each relative type was coded as a multilevel categorical time-varying variable to investigate the association between the number of each relative type and child mortality. In the variable, each class indicates the number of relatives of a given type who were simultaneously alive at each age of the child, based on their exact or censored lifespan. As the distributions of the number of relatives of a given type are right-skewed (because only a small number of children had very high numbers of each type of kin present at the same time), we chose to use a multilevel categorical variable instead of a continuous variable for the presence of each relative type.

First, we conducted discrete time-event analyses, which allowed us to analyse the time-dependent presence of each relative type on child mortality at each age until five years. We ran separate analyses for each relative type, with the same set of confounding variables that are known to be associated with child survival (Chapman et al., 2021; Lahdenperä et al., 2011). The fixed variables were: child age (linear and quadratic), sex, twin (yes/no), birth year (continuous), birth order (categorical: 1/2–4/≥ 5), mother’s age (linear and quadratic), family SES (high/moderate/low) and living region. The quadratic terms of child age and maternal age were selected based on model fit and as the mortality risk decreases exponentially with age after birth (Engelman et al., 2017) and both young and advanced maternal age is associated with adverse birth and child outcomes (e.g. Fall et al., 2015). As the mother’s presence is also known to strongly associate with child mortality during the first years of life, we included the mother’s presence in all models (Lahdenperä et al., 2011). The mother’s identity was included as a repeated variable to take into account the interdependency of survival between children in the same family. For each relative type, the odds ratios (ORs) and their 95% confidence limits (CLs) were calculated to represent the increase in the average risk of mortality with the increasing number of relatives present as compared to zero relatives of this type present. We assessed age-specific mortality using generalised linear mixed models (GLMMs) with binomial errors and a logit link function with a GENMOD procedure in SAS (SAS Institute Inc., release 9.4, 2014).

Second, with similar methods, we ran models where the presence of each relative was coded as a binomial variable, 0, when no given type of relative were alive and, 1, when at least one given type of relative was alive. These models were used to test interactions with child age or family SES and the presence of each relative type, as the associations with child mortality may vary with the care dependency of children and the amount of available resources in the family. Interactions with child sex and the presence of each relative type were also originally tested, but removed from final analyses as no significant interactions were observed. The two-level categorical variable of each relative type was used in these models instead of multilevel categorical variables to increase the power to detect statistically significant interactions and to ease the interpretation of the associations.

Third, we calculated the number of each type of relative who had died by each age of the child and whose survival information was missing at each age (i.e. were censored already before the child was born or before the child was five years old and thus their presence was unknown). To test the effect of missing information on the robustness of the results, we ran additional models with similar methods (with a multilevel categorical variable and a binomial variable for the presence of each relative type). In these models, we used a time-varying covariate of each relative type which measured the percentage of relatives that had certain information about their presence at each age.

In summary, the main results are reported from models where a multilevel categorical variable of each relative type was used, whereas the results for interactions with child age and family SES are reported from models where a binomial variable of each relative type was used. A robustness check is reported from models where the percentage of missing information for the presence of each relative type has been used as a covariate.

## 3. Results

### 3.1 Characteristics of the children and families in historical Finland

In general, 73% of children survived to age 5 (Table 1). The survival was associated with child sex, twin status, birth order, mother’s age at birth, family SES, and living region that were controlled for in all models (Table 1). Particularly, boys, twins, firstborns, children from poorer families (low SES), children born to younger and older mothers, and children from eastern or northern Finland had lower survival to age 5 than other children.

Almost all children had both parents present (alive; 99.9%) and 75.7% had at least one sibling present at birth (Supplementary Table S1). Nearly half of the children had a grandparent present at birth (paternal grandmother (52%), paternal grandfather (39%), maternal grandmother (58%), maternal grandfather present (43%)). Paternal aunt(s) or uncle(s) was present at birth for 72–74% of children and from the maternal side for 73–76% of children. Of more distant relatives, 70–72% of children had paternal or maternal cousin(s) present at birth, 48–57% had a great-aunt or great-uncle present, and only 6–8% had a great-grandparent present (Supplementary Table S1). The great-grandparents were excluded from subsequent child survival analyses, as their presence was relatively rare at birth (2–8%). The percentage of children having at least one same-generation relative present increased with age as more of them were born (e.g. siblings and cousins, Supplementary Tables S1, S2, Figure S1), whereas the percentage of children having at least one older-generation relative (e.g. parents, aunts/uncles, great-aunts/great-uncles, grandparents) decreased between birth and age 5 (Supplementary Tables S1, S2, Figure S1). Missing information was relatively rare for closer relatives (1–2%) but more common for more distant relatives (2–28%), being highest for great-aunts or -uncles (Supplementary Table S1).

### 3.2 Presence of relatives and child mortality

#### 3.2.1 Close family members

**Figure 1.**
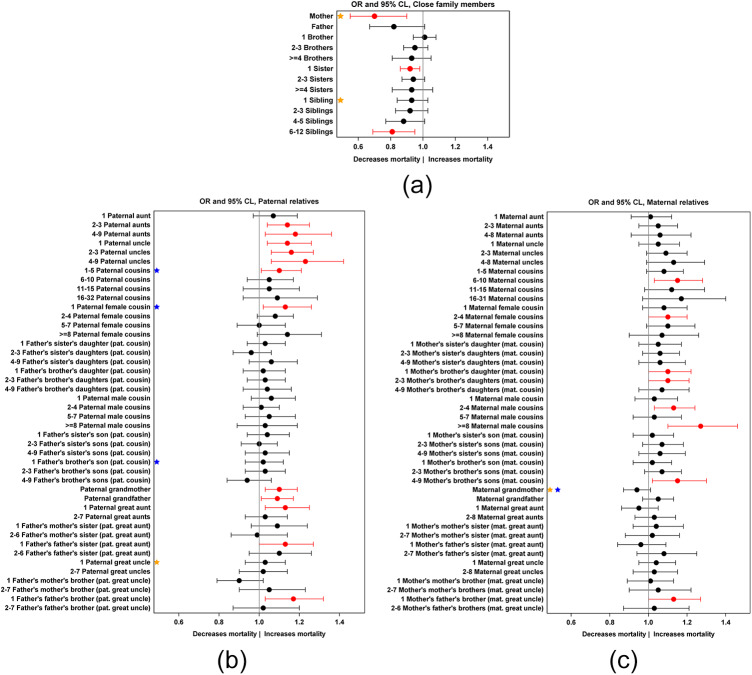
Odds ratios of child mortality from birth to 5 years by the number of each relative type being alive. An odds ratio higher than 1.0 indicates increased mortality before age 5 and lower than 1.0 decreased mortality with the presence/number of each relative type. Child mortality risk and presence of (a) close family members, (b) paternal relatives, (c) maternal relatives. The presence of the relative has been coded as a binary variable (0/1) for mother, father, and grandparents, and for all other relative types as a multilevel variable by the number of relatives being alive simultaneously. The significances of the main term (each relative type) from multilevel models are given in Supplementary Table S3. Red values with 95% confidence limits (CLs) show significant associations from pairwise comparisons with 0 (i.e. no living relative of this specific type) as the reference category, and black indicates non-significant associations. Thus, the figures show how the increasing number of each relative type is associated with child mortality risk. Stars depict interactive associations, with orange stars showing significant associations with the relative type and child age and blue stars show significant associations with the relative type and family SES. In the interaction models the presence of each relative type has been coded as a binomial variable (0/1, i.e. no relative alive vs. at least 1 alive) (Supplementary Table S4).

First, the mother’s presence was crucial for child survival during the first five years of life as it decreased odds of child mortality substantially, by 30% (OR (95% CLs): 0.70 (0.55, 0.90), *p* = 0.01; Supplementary Table S3, Figure 1a). The mother’s presence was associated with the greatest decrease in child mortality before the age of 2 years (*p*(mother × child age interaction) = 0.02, Supplementary Table S4, Figure S2a). The presence of the father had a decreasing but non-significant association with child mortality (OR: 0.82 (0.67, 1.01), *p* = 0.08; Supplementary Table S3, Figure 1a). However, only less than 3% of children lost their mother and about 4% lost their father before the age of 5 years (Supplementary Table S2). The presence of siblings, both sisters and brothers, had non-significant associations with child mortality risk until 5 years of age (Supplementary Table S3, Figure 1a), but pairwise comparisons showed that there are some significant associations between the number of these relatives and the mortality risk of the child. The presence of one living sister (29% of children at birth) was associated with a decrease in the odds of child mortality of 8% compared to children who had no sisters present (43% of children at birth) (OR: 0.92 (0.86, 0.98), *p* = 0.01; Supplementary Table S3, Figure 1a) and the increasing number of living sisters (28% of children had > 1 sister at birth) had very similar associations with child mortality risk, although these other comparisons were statistically non-significant. Similarly, the increasing number of present siblings had an intensifying association with child mortality but only when there were more than 6–12 present siblings (6% of children at birth) did the odds of child mortality risk decrease statistically significantly, by 19%, compared to children who had no siblings present (24% of children at birth) (OR: 0.81 (0.69, 0.95), *p* = 0.01; Supplementary Table S3, Figure 1a). The association between the presence of siblings and child mortality also depended on the child’s age, and the presence of siblings was associated with a slight decrease in child mortality before age one (*p*(siblings × child age interaction) = 0.006, Supplementary Table S4, Figure S2b). The associations with close family members and child mortality were not dependent on the family SES (*p*-values for all interactions > 0.05, Supplementary Table S4).

#### 3.2.2 Paternal relatives

The presence of paternal aunts (*p* = 0.02) and uncles (*p* = 0.006) was associated with a substantial increase in child mortality risk before age 5 (Supplementary Table S3, Figure 1b). There was a linear increase in the associations with a higher number of these relatives being linked to an increased mortality risk of children. The mortality risk was no higher for children with only one paternal aunt (27% of children at birth; *p* = 0.16), whereas children with only one paternal uncle had 14% higher odds of mortality (29% of children at birth; OR: 1.14 (1.04, 1.26), *p* = 0.002), and children with more than four paternal aunts (10% of children at birth) or uncles (8% of children at birth) had 18% and 23% higher odds of mortality, respectively, compared to children without any paternal aunts (25% of children at birth) or uncles (28% of children at birth) (aunts: OR: 1.18 (1.03, 1.36), *p* = 0.02; uncles: OR: 1.23 (1.06, 1.42), *p* = 0.005; Supplementary Table S3, Figure 1a). Paternal cousins, divided by sex (paternal female cousins and paternal male cousins) or, specifically, divided into father’s sister’s children and father’s brother’s children, had no significant main associations with child mortality risk (Supplementary Table S3, Figure 1b). However, pairwise comparisons showed that children with 1–5 paternal cousins present (38% of children at birth; OR: 1.10 (1.01, 1.21), *p* = 0.03; Supplementary Table S3, Figure 1b) or one paternal female cousin (15% of children at birth; OR: 1.13 (1.02, 1.26), *p* = 0.02; Supplementary Table S3, Figure 1b) had slightly increased odds of mortality compared to children with no paternal cousins (28% of children at birth) or paternal female cousins (36% of children at birth). The mortality risk had no increasing associations with a higher number of any type of paternal cousins. Instead, the effect of paternal cousins on child mortality depended on the family SES: the presence of any paternal cousins (*p*(presence × family SES interaction) = 0.03), paternal female cousins (*p*(interaction) = 0.03), or specifically, father’s brother’s son (*p*(interaction) = 0.04), was associated with an increase in the child’s odds of mortality by 20%, 18%, and 10%, respectively, among the high SES group but not among those of moderate or low SES groups (Supplementary Table S4, Figure S3a–c).

The presence of paternal grandmother (52% of children at birth) and grandfather (39% of children at birth) was associated with an increase in the odds of child mortality before the age of 5 by 10% and 9%, respectively (grandmother: OR(95% CLs): 1.10 (1.03, 1.19), *p* = 0.008; grandfather: OR(95% CLs): 1.09 (1.01, 1.17), *p* = 0.02; Supplementary Table S3, Figure 1b). The presence of paternal great-aunts (*p* = 0.04), particularly having one paternal great-aunt (28% of children at birth; *p* = 0.01), was associated with an increase in the odds of child mortality by 13% compared to children who had no paternal great-aunts present (46% of children at birth; OR: 1.13 (1.03, 1.25), Supplementary Table S3, Figure 1b). The presence of paternal great-uncles (48% of children at birth) was associated with an increase in the odds of child mortality before the age of 1 year (*p*(interaction) = 0.03, Supplementary Table S4, Figure S2c). Moreover, father’s father’s brothers (*p* = 0.05), particularly having one paternal great-uncle (25% of children at birth; *p* = 0.01), were associated with an increase in the odds of child mortality by 17% compared to children who had no father’s father’s brothers present (62% of children at birth; OR: 1.17 (1.03, 1.32), Supplementary Table S3, Figure 1b).

#### 3.2.3 Maternal relatives

Maternal aunts’ and uncles’ presence (76% and 73% of children at birth, respectively) was not associated with child mortality risk until 5 years of age (Supplementary Table S3, Table S4, Figure 1c). Comparisons between multilevel categories of maternal cousins showed that having 6–10 maternal cousins present (19% of children at birth) was associated with an increase in the odds of child mortality by 15% (OR: 1.15 (1.03, 1.28), *p* = 0.01) compared to children that had no maternal cousins present (30% of children at birth), and the increasing number of maternal cousins had a similar association with child mortality, although non-significantly (11–15 maternal cousins (OR): 1.12 (0.98, 1.29), *p* = 0.09; 16–31 maternal cousins (OR): 1.17 (0.97, 1.40), *p* = 0.09, Supplementary Table S3, Figure 1c). In particular, maternal male cousins had an increasing association with child mortality (*p* = 0.005) and pairwise comparisons showed increased odds for mortality when 2–4 (29% of children at birth; OR: 1.13 (1.03, 1.24), *p* = 0.007) or more than 8 maternal male cousins were present (6% of children at birth; OR: 1.23 (1.06, 1.42), *p* = 0.001, Supplementary Table S3, Figure 1c). More specifically, among maternal male cousins, the presence of 4–9 mother’s brother’s sons (10% of children) was associated with an increase in the odds of child mortality by 15% (OR: 1.15 (1.02, 1.30), *p* = 0.02, Supplementary Table S3, Figure 1c). Furthermore, the presence of 2–4 maternal female cousins (29% of children at birth) was associated with an increase in the odds of child mortality by 10% (OR: 1.10 (1.00, 1.20), *p* = 0.05), which again may have derived from the presence of mother’s brother’s daughters, as the presence of 2–3 of them (18% of children at birth) was associated with an increase in the odds of child mortality by 10% (OR: 1.10 (1.00, 1.21), *p* = 0.05, Supplementary Table S3, Figure 1c). Mother’s brother’s daughter’s association with child mortality also depended on the age of the child (*p* = 0.03, Supplementary Table S4) and their presence (41% of children at birth) was associated with a slight increase in child mortality before the age of 2 years (Supplementary Figure S2d). The presence of maternal aunts, uncles, or other types of maternal cousins had no statistically significant interacting associations with child age or family SES in their effects on child mortality (Supplementary Table S3).

Maternal grandmother’s association with child mortality depended on the age of the child (*p*(interaction) = 0.03, Supplementary Table S4) and the family SES (*p*(interaction) = 0.009, Supplementary Table S4). The presence of maternal grandmother (58% of children at birth) was associated with a slight decrease in child mortality after the age of 2 years (Supplementary Figure S2e). The presence of the maternal grandmother was associated with a decrease in child mortality among the moderate and low SES families, but not significantly so among the high SES families, as their odds of mortality decreased by 17% (OR: 0.83 (0.74, 0.94)) and 14% (OR: 0.86 (0.74, 1.01)), respectively (Supplementary Figure S3d). Maternal grandfather’s presence (43% of children at birth) had no significant association with child mortality (Supplementary Tables S3, Figure 1c). Also, the presence of maternal great-aunts (57% of children at birth) and great-uncles (50% of children at birth) of any type were not associated with child mortality (Supplementary Table S3, interactions with age and family SES: Supplementary Table S4), except the presence of one mother’s father’s brother (25% of children at birth) was associated with an increase in the odds of child mortality of 13% compared to children who had no mother’s father’s brothers present (58% of children; OR: 1.13 (1.00, 1.27), *p* = 0.05, Supplementary Table S3, Figure 1c).

The results from each type of relative presence on child mortality remained similar in models where the percentage of missing information on the presence of each type of relative is adjusted for in the analyses (multilevel categorical models, Supplementary Table S5; binomial models with interactions between relative type and age or family SES, Supplementary Table S6).

## 4. Discussion

A variety of kin contribute to childcare and their presence has been shown to be associated with child mortality in numerous traditional human societies. However, the specific effects of close and more distant types of relatives have rarely been investigated simultaneously in the same study setting. Our study revealed complex associations between these relatives. We found that the presence and number of several different types of relatives were associated with child mortality risk in historical Finland. The presence of certain relatives was associated with a decrease in child mortality, whereas some other relatives’ presence was associated with an increase in it. In addition, the magnitude of the associations varied between different types of relatives and many of the associations were dependent on the lineage, child age, or family SES, as we predicted. We discuss the findings in the light of kin selection theory, resource competition theory, intergenerational transfer theory, and sex-specific reproductive strategies.

First, in line with our first prediction and kin selection theory (Hamilton, 1964), we found that more closely related individuals were associated with the greatest decreases in child mortality. The mother’s presence was most crucial for the infant, especially during the first 2 years of life. This is not surprising, as other family members can hardly compensate for the loss of the mother due to breastfeeding in the first years of life (Lahdenperä et al., 2011). However, the loss of a mother was a relatively rare event, and concerned less than 3% of children. In addition, the presence of a sister or several siblings (6–12) was associated with a decrease in child mortality. This may be due to the sister and siblings, especially in larger families, actively taking part in child rearing, which has been reported to occur in several other traditional populations (Beise, 2005; Crognier et al., 2001, 2002; Nitsch et al., 2013; Sear, 2008; Sear et al., 2002). About one-third of children had a sister present, whereas only about 6–9% of children had 6–12 siblings present before the age of 5 years, suggesting that the sister’s presence was of greater importance in this population. However, the association of siblings with child mortality depended on the child’s age, and the presence of any siblings (76% of children at birth) had a slightly decreasing association during the first year of the child’s life, suggesting, somewhat surprisingly, that their help may be of particular importance during infancy. It is noteworthy that our findings of the presence of siblings (and sisters and brothers) on child mortality may have been underestimated. This is likely because our measure of sibling presence included both full (relatedness coefficient 0.50) and half-siblings (relatedness coefficient 0.25). Half-siblings have been shown to have more conflicts than full siblings (Tanskanen et al., 2017), and sometimes the associations have been negative for the child’s well-being (Strow & Strow, 2008). Moreover, we did not control for the interbirth-interval length between the previous or the subsequent sibling in the family in the models, as we were interested in how the number of siblings is associated with child mortality risk. However, it has been shown that children with short birth intervals might have had increased mortality risk (Blurton Jones, 2016; Islam et al., 2022; Lahdenperä et al., 2011). Finally, we did not separate older and younger siblings in our analyses although their associations with child survival may differ. Older siblings are more likely to be helpful and younger siblings are more likely to be competitors for parental care (Nitsch et al., 2013; Sear & Coall, 2011). The father’s and brothers’ presences were not significantly associated with child mortality, in line with a previous study in historical Finland (Lahdenperä et al., 2011), although the father’s presence was associated with a decreasing trend on mortality.

Second, we found that the presence of paternal relatives was associated with an increase in child mortality more often than the presence of maternal relatives, in line with more intense resource competition with paternal kin in this patrilocal society (Chapman et al., 2021, 2019; Lahdenperä et al., 2012; Nitsch et al., 2014; Pettay et al., 2016). In fact, we did not detect a decrease in child mortality associated with the presence of paternal relatives of any kind. We found that the presence of paternal aunts and uncles was particularly detrimental for the child: the presence of each aunt or uncle was further associated with an increase in child mortality. Almost 72% and 75% of children had at least one paternal aunt or uncle present at birth and 48% and 43% had more than one, making the presence of these relatives particularly important in this population. Our finding is consistent with a previous study in the same population showing that the presence of paternal aunts and uncles was associated with lower child survival (Nitsch et al., 2014). Compared to other populations, our finding is in line with a study from an eighteenth- to nineteenth-century East Asian population, where paternal aunts and uncles decreased child survival (Dong et al., 2017) but in contrast with a study from a nineteenth-century American frontier population, where the presence of paternal aunts was associated with increased infant survival (Heath, 2003). Moreover, some studies have found that the associations between the presence of aunts and uncles and child survival might be linked to resource inheritance patterns. In patrilocal Kipsigis of Kenya, paternal uncles decreased child mortality particularly in wealthier families (Borgerhoff Mulder, 2007), but in rural Malawi detrimental effects of aunts on child survival were linked with resource inheritance patterns and competition (Sear, 2008). In patrilocal historical Finland, transfer of land on the male line favoured the eldest and co-residence with paternal relatives was common. In some areas in Finland, in addition to extended households with the oldest married son and paternal grandparents, joint residence by married sons or siblings was common (Moring, 1998). Therefore, the higher number of paternal siblings often meant higher competition for the shared resources within the same household (Pettay et al., 2016). Also, 72–77% of children had at least one paternal cousin present before the age of 5 years and their presence (particularly paternal female cousins and father’s brother’s sons) was associated with an increase in child mortality among the high SES families but not among the moderate or low SES families, giving further support that these associations may have been more intense in those with more resources. In line with a previous study from the same population (Chapman et al., 2019) and some other historical patrilocal populations (Beise & Voland, 2002; Jamison et al., 2002; Voland & Beise, 2002) we also found that the presence of paternal grandmother and paternal grandfather (Campbell & Lee, 1996; Derosas, 2002; Kemkes-Grottenthaler, 2005) were associated with an increase in child mortality, which may also result from resource competition in shared households (Kemkes-Grottenthaler, 2005; Lahdenperä et al., 2012). Moreover, paternal grandparents were usually a few years older than maternal grandparents and may have been in poorer health, acting as competitors with the child for parental resources (Chapman et al., 2019). Interestingly, the presence of paternal great-aunts and -uncles, particularly from the grandfather’s side, were also associated with an increase in child mortality risk. About half of the children had at least one paternal great-aunt or -uncle present before the age of 5 years, suggesting that their presence was important for the lives of children in historical Finland. This association may be explained by the household structure, as in horizontally extended families, older paternal relatives (particularly uncles) also generally stayed in the household all their lives (Moring, 1998). This may explain why the presence of these specific relatives, potentially also needing care when older, could have negative associations with child survival.

Third, in contrast to our second prediction that maternal relatives reduce child mortality more than paternal relatives, we found that many types of maternal relatives were also associated with increased child mortality risk. This is in contrast with the cross-cultural matrilateral bias in alloparenting (Perry & Daly, 2017). The presence and number of maternal aunts and uncles were not associated with child mortality risk, in line with our previous study in historical Finland (Nitsch et al., 2014), but in contrast to a previous study from historical American population, where maternal aunts and uncles decreased child mortality (Heath, 2003). However, the presence of their children was. The presence of a higher number of maternal cousins (in particular six or more maternal cousins) were associated with increased child mortality. Before the age of 5 years, 33–40% of children had more than six maternal cousins present, indicating the relative importance of these findings. Especially, a higher number of maternal male cousins (mother’s brother’s sons) was associated with increased child mortality. However, there was also some indication that a higher number of maternal female cousins (mother’s brother’s daughters) was associated with increased child mortality. There is a clear lack of studies investigating how the presence of cousins, either from the maternal or paternal side, is associated with child mortality. However, one recent study shows that in an early twentieth-century North American population the higher number of maternal relatives (combining maternal uncles, aunts, nephews, nieces, and cousins into one measure) decreased child mortality (Harton et al., 2023), which is in contrast to our findings and suggests that the associations may be population-specific, or dependent on the particular relative type. In fact, our results concerning maternal cousins are also likely to differ from the resource-linked paternal cousin associations with child mortality. Although in some areas of historical Finland, sisters’ and brothers’ families could also share the same household, this had already became relatively rare in the nineteeth century (Moring, 1998). Therefore, although there could have been some degree of resource competition between the mother’s siblings’ families, especially in the case of mother’s brothers’ children, it is not likely to explain all cousin associations, as the presence of maternal aunts and uncles per se was not associated with higher child mortality (in comparison to paternal aunts and uncles). A more likely explanation is that the maternal cousins were competing for the same maternal grandmother’s attention and investment in grandchildren, which was potentially diffused across all children and grandchildren when more of them were present (Coall et al., 2009; Danielsbacka, 2016).

Indeed, we found that maternal grandmothers were important and their presence was associated with decreased child mortality, but the association depended on the child’s age and the family SES. These findings have biological meaning, as 50–58% of children had a maternal grandmother present before the age of 5 years. Maternal grandmother’s presence was associated with a slight decrease in child survival after infancy and the first year of the child’s life, along the lines of previous studies in this population (Chapman et al., 2019, 2021) and many other historical and contemporary populations (Beise, 2005; Beise & Voland, 2002; Heath, 2003; Ragsdale, 2004; Sear & Coall, 2011; Sear & Mace, 2008; Sear et al., 2002; Tymicki, 2009; Voland & Beise, 2002). However, the most substantial association between the maternal grandmother and child mortality was seen among the moderate SES families, in which odds for child mortality were 17% lower if the grandmother was alive. A decreasing trend was also observed among the low SES families, whose children’s odds for mortality decreased by 14% in the presence of a maternal grandmother. This decrease among moderate SES families led to significantly lower mortality of children compared to mortality of children among high SES families with maternal grandmother present (*p* = 0.01), suggesting maternal grandmother’s help was not only compensating for fewer resources but also brought additional benefits to the families. This was not the case among low SES families, where the maternal grandmother’s presence led to similar but not significantly lower mortality rates as in high SES families (*p* = 0.55). However, the maternal grandmother’s presence was associated with a decrease in child mortality among the moderate and low SES families the most after the mother’s presence (which reduced odds for mortality by 30%), and the effect size is greater than, for example, the presence of a sister (reduced odds for mortality by 8%). Our results are consistent with a previous study in historical Bohemia (Czech Republic), where grandmothers’ presence increased the survival probability of children up to 5 years of age only in families with the lowest SES (lodgers) (Havlíček et al., 2021). On the contrary, some studies have found that when resources are scarce, the presence of a grandmother has been associated with increased child mortality (Borgerhoff Mulder, 2007; Strassmann & Garrard, 2011). Our result is likely to arise from household structure, the amount of available resources, and the need for grandmother’s assistance. In our study population, most of the high SES families were landowners, whose typical households included several non-family members such as servants, who usually assisted in diverse farming duties but could also take part in childcare (Moring, 2003). These families most likely included paternal grandmothers in the same household as a result of the inheritance system, diminishing the importance of maternal grandmothers. In contrast, the moderate and low SES families included tenant farmers, craftsmen, fishermen and other farmless families and servants, respectively, and were potentially more dependent on the maternal grandmother’s help in childcare in the absence of other helpers. In addition to childcare, any other help from maternal grandmothers to the parents or provision of resources could explain these associations among lower SES families. Also, child mortality was higher among the low SES families, as approximately 30% of children died before age 5 in this study population, and thus there was a higher potential for grandmothers to have a meaningful impact on grandchild survival.

To summarise, our findings are consistent with our third prediction concerning sex-specific reproductive strategies where women invest more in children than men (Trivers, 1972), as only the presence of female relatives was associated with a decrease in child mortality. Also, empirical evidence from other populations is consistent with this finding (e.g. Sear & Coall, 2011; Sear & Mace, 2008). However, we found both negative and positive associations with the presence of relatives on child mortality in the older and the same generation, although we predicted that there would be more competitive associations between relatives from the same generation in line with the resource competition hypothesis (Nitsch et al., 2013) and more helping associations in the older generations in line with the intergenerational transfer theory (Lee, 2003, 2008). Our findings are likely to be explained by the complex modifying associations with the family SES and available resources, as well as household composition, making paternal relatives more likely competitors for resources and maternal relatives more likely helpers who did not compete for the same resources within the household. Our results, however, indicate that maternal relatives, especially from the same generation, may act as competitors (e.g. from maternal grandmother’s care), but probably for different reasons than paternal relatives (resource competition).

There are some important issues that need to be considered when interpreting the results and implications of our study. The survival status of relatives has often been used as a proxy for the availability of relatives to help mothers, although it does not include information on the actual interaction rates between relatives and it is possible that shared genes or shared environment might result in positive associations between the survival of children and their relatives. However, we found both positive and negative associations with the relatives’ presence, indicating the results are not solely due to increased lifespan in specific families. We also controlled for a wide set of variables, such as living area, family SES, and shared maternal effects, making it unlikely that shared family or environmental factors would explain our results. Even so, when investigating associations between the survival status of the relative (i.e., presence in our study) and child mortality we are not directly measuring causality, and these associations remain an indirect estimate of the actual relationship (Coall et al., 2018; Helle et al., 2024). Furthermore, our measure of kin presence may underestimate our findings on the effect of different kin on child mortality. We represented kin presence by the number of relatives of each type who were certainly alive simultaneously with the child. Thus, the reference category in calculating the odds ratios was having no living relative of that type. However, in some cases these relatives could have been alive but were censored. For most relative types, the missing information concerned less than 10% of the cases, but in the case of (great)aunts and (great)uncles the uncertainty percentages were substantially greater. Nevertheless, there are two reasons why this missing information is unlikely to bias our results. First, it is most likely that we have missing information for the relative’s lifespan if they dispersed from the natal region to some other region or abroad and could not be followed in the church registers. In most of these cases, the relative would not have had a high contact rate with the family and was unlikely to have made a difference in child mortality. Second, when we included the percentage of missing information as a covariate in the models, the results remained similar and robust.

Furthermore, our analyses did not take into account each relative’s age, as there were several of them of different ages in most cases. The age-dependent effects of the relatives may have thus confounded our analyses, as the effects of different-aged relatives may have cancelled each other out (Chapman et al., 2019). In addition, our study approach included a large number of analyses, as each relative type needed separate analyses due to differing sample sizes, and we did not use adjustments for multiple testing. However, it should be noted that the use of corrections has been also debated (Rothman, 1990). Lastly, our analyses leave all the other effects of relatives on child well-being unexplored, which might also be important, as we investigated the extreme measure of child well-being in terms of mortality. There is a large amount of research showing, on the one hand, human children are well adapted to having non-parental helpers and they have positive effects on children’s growth, development, education, or physical and psychological well-being (Sear & Coall, 2011; Tanskanen & Danielsbacka, 2018). For example, during the demographic transition in Spain, even though fathers had little effect on the survival of their young children, teenage boys had shorter stature in the absence of fathers (Reher & González-Quinones, 2003). On the other hand, similarly to all other social relationships, interactions with kin may increase the risk of social transmission of infectious diseases, which may or may not be shown as lower child survival (Kappeler et al., 2015). It was recently shown that, in pre-industrial Finland, at least some of the maternal grandmother effects may have been mediated through decreased mortality to smallpox and pulmonary and diarrhoeal infections (Ukonaho et al., 2023). Although our results are the first to show comparable associations between the closer and more distant relatives on child mortality in the same study setting, more studies are needed to clarify the complex associations of relatives in different societies, cultures, (co)residence patterns, and socioeconomic groups.

In conclusion, our results show that in historical Finland, the presence of many relatives increased child mortality. The effects of paternal relatives were particularly strong among the high SES families, as their family configuration was likely to involve more intense competition for common resources. On the contrary, the maternal grandmother was observed to decrease child mortality the most among the moderate and low SES families, who probably needed the grandmother’s contribution to childcare more than the high SES families. Our results bring new insights into the importance of close and more distant kin and suggest that relatives can provide support or other resources but also spread diseases (Kappeler et al., 2015; Ketola et al., 2021) and compete for limited resources and care. Although the nuclear family is the norm today in developed countries such as Finland, it is the exception historically and in many other cultural contexts (Kramer, 2021). Thus, by investigating the benefits and costs of the presence of all relatives in various contexts, it becomes possible to enhance our understanding of the limitations and possibilities that arise from the organisation of nuclear families. Clarifying how the presence of relatives is associated with child mortality in these historical contexts can offer a broader perspective of human family life and also give important insights into the evolution of family, cooperative breeding, and conflicting interactions in humans.

## Supporting information

Lahdenperä et al. supplementary material Lahdenperä et al. supplementary material

## Supplementary material

The supplementary material for this article can be found at https://doi.org/10.1017/ehs.2024.47.
