# Supplementary material for: Close and more distant relatives are associated with child mortality risk in historical Finland
Source: Evol Hum Sci. 2025 Jan 20;7:e5. doi: 10.1017/ehs.2024.47 (PMC11811707; doi:10.1017/ehs.2024.47)
Supplement: Lahdenperä et al. supplementary material [file S2513843X24000471sup001.docx]

**Supplementary material**

**Close and more distant relatives are associated with child mortality risk in historical Finland**

**Lahdenperä et al.**

**Table S1.** Presence of relatives at birth and age 5. Presence % indicates the percentage of the children that had at least one relative of the given type alive at birth or at 5 years of age. Percentages are also given for the children that had missing information on the presence of at least one given type of relative at birth. Relatives with less than 10 % presence at birth were not investigated in child survival models.

| *Relative* | N (children) | Range of relatives present at birth | % with ≥1 relative present at birth | % with ≥1 relative present at age 5 | % with ≥1 relative missing at birth |
| --- | --- | --- | --- | --- | --- |
| Mother | 31392 | 0-1 | 100.00 | 97.25 | - |
| Father | 31392 | 0-1 | 99.92 | 95.91 | - |
| Brother | 31392 | 0-8 | 57.66 | 74.66 | 1.09 |
| Sisters | 31392 | 0-9 | 57.20 | 74.49 | 1.11 |
| Siblings | 31392 | 0-12 | 75.70 | 94.25 | 1.98 |
| *Paternal relatives* |  |  |  |  |  |
| Paternal aunt | 16407 | 0-9 | 74.71 | 71.92 | 15.24 |
| Paternal uncle | 16407 | 0-7 | 72.19 | 69.24 | 14.34 |
| Paternal cousins | 16407 | 0-30 | 72.09 | 77.28 | 9.73 |
| Paternal female cousins | 16407 | 0-16 | 63.75 | 69.88 | 6.49 |
| Father’s sister’s daughter | 16407 | 0-9 | 47.31 | 52.38 | 4.08 |
| Father’s brother’s daughter | 16407 | 0-9 | 40.57 | 46.12 | 2.87 |
| Paternal male cousins | 16407 | 0-18 | 64.33 | 70.50 | 5.93 |
| Father’s sister’s son | 16407 | 0-9 | 47.84 | 52.86 | 3.57 |
| Father’s brother’s son | 16407 | 0-9 | 40.42 | 46.57 | 2.71 |
| Paternal grandmother | 16379 | 0-1 | 52.27 | 43.32 | 4.08 |
| Paternal grandfather | 16138 | 0-1 | 38.86 | 30.23 | 3.48 |
| Paternal great-aunt | 11480 | 0-7 | 54.01 | 48.55 | 27.60 |
| Father’s mother’s sister | 7240 | 0-6 | 49.02 | 44.03 | 25.18 |
| Father’s_father’s_sister | 7096 | 0-6 | 43.77 | 38.51 | 21.41 |
| Paternal great-uncle | 11480 | 0-7 | 47.77 | 42.12 | 23.42 |
| Father’s_mother’s_brother | 7240 | 0-7 | 43.38 | 38.88 | 18.25 |
| Father’s father’s brother | 7096 | 0-6 | 38.32 | 32.73 | 20.14 |
| Paternal great-grandparents | 11480 | 0-3 | 5.59 | 2.82 | 10.72 |
| Father’s mother’s mother | 7240 | 0-1 | 3.95 | 1.92 | 9.01 |
| Father’s father’s mother | 7083 | 0-1 | 2.85 | 1.30 | 9.88 |
| Father’s mother’s father | 7179 | 0-1 | 1.81 | 0.75 | 7.42 |
| Father’s father’s father | 7050 | 0-1 | 1.69 | 1.01 | 7.69 |
| *Maternal relatives* |  |  |  |  |  |
| Maternal aunt | 16844 | 0-8 | 76.19 | 74.41 | 13.58 |
| Maternal uncle | 16844 | 0-8 | 73.21 | 71.20 | 12.40 |
| Maternal cousins | 16844 | 0-31 | 70.20 | 77.05 | 7.58 |
| Maternal female cousins | 16844 | 0-17 | 61.81 | 69.52 | 5.40 |
| Mother’s sister’s daughter | 16844 | 0-9 | 44.37 | 50.83 | 3.82 |
| Mother’s brother’s daughter | 16844 | 0-9 | 40.95 | 47.00 | 2.07 |
| Maternal male cousins | 16844 | 0-18 | 62.32 | 69.55 | 4.29 |
| Mother’s sister’s son | 16844 | 0-9 | 45.28 | 51.05 | 2.78 |
| Mother’s brother’s son | 16844 | 0-9 | 41.36 | 47.78 | 1.78 |
| Maternal grandmother | 16812 | 0-1 | 57.57 | 49.82 | 4.55 |
| Maternal grandfather | 16584 | 0-1 | 43.40 | 34.95 | 3.09 |
| Maternal great-aunt | 12335 | 0-8 | 56.89 | 51.83 | 25.87 |
| Mother’s mother’s sister | 7535 | 0-7 | 50.82 | 46.11 | 21.74 |
| Mother’s father’s sister | 7679 | 0-7 | 47.71 | 42.83 | 21.66 |
| Maternal great-uncle | 12335 | 0-8 | 50.90 | 45.79 | 22.69 |
| Mother’s mother’s brother | 7535 | 0-7 | 45.84 | 40.99 | 16.99 |
| Mother’s father’s brother | 7679 | 0-6 | 42.30 | 37.45 | 20.50 |
| Maternal great-grandparents | 12335 | 0-3 | 7.81 | 4.22 | 10.76 |
| Mother’s mother’s mother | 7535 | 0-1 | 6.12 | 3.45 | 9.49 |
| Mother’s father’s mother | 7640 | 0-1 | 3.85 | 1.92 | 9.86 |
| Mother’s mother’s father | 7430 | 0-1 | 2.85 | 1.42 | 7.09 |
| Mother’s father’s father | 7646 | 0-1 | 1.84 | 0.85 | 8.24 |

**Table S2.** Presence of relatives (by multilevel categorical variable) each year from birth to age 5. Sample size, n, indicates the number of children and % indicates the percentage of the children in each category.

| **Relative** | **Number present** | **Age 0, n(%)** | **Age 1, n(%)** | **Age 2, n(%)** | **Age 3, n(%)** | **Age 4, n(%)** |
| --- | --- | --- | --- | --- | --- | --- |
| Mother | 0 | 0 (0) | 147 (0.5) | 257 (1.0) | 446 (1.8) | 649 (2.8) |
|  | 1 | 31,392 (100%) | 27,097 (99.5) | 25,190 (99.0) | 23,904 (98.2) | 22,925 (97.2) |
| Father | 0 | 26 (0.1) | 261 (1.0) | 475 (1.9) | 697 (2.9) | 964 (4.1) |
|  | 1 | 31,366 (99.9) | 26,983 (99.0) | 24,972 (98.1) | 23,653 (97.1) | 22610 (95.9) |
| Brother | 0 | 13,291 (42.3) | 11,378 (41.8) | 8,836 (34.7) | 6,869 (28.2) | 5,974 (25.3) |
|  | 1 | 9,257 (29.5) | 8,291 (30.4) | 8,537 (33.6) | 8,557 (35.1) | 8,187 (34.7) |
|  | 2-3 | 7,450 (23.7) | 6,404 (23.5) | 6,802 (26.7) | 7,472 (30.7) | 7,870 (33.4) |
|  | 4-8 | 1,394 (4.4) | 1,171 (4.3) | 1,272 (5.0) | 1,452 (6.0) | 1,543 (6.6) |
| Sisters | 0 | 13,436 (42.8) | 11,456 (42.0) | 8,987 (35.2) | 6,979 (28.7) | 6,013 (25.5) |
|  | 1 | 9,039 (28.8) | 8,224 (30.2) | 8,469 (33.3) | 8,556 (35.1) | 8,259 (35.0) |
|  | 2-3 | 7,616 (24.3) | 6,476 (23.8) | 6,780 (26.6) | 7,410 (30.4) | 7,832 (33.2) |
|  | 4-9 | 1,301 (4.1) | 1,088 (4.00) | 1,211 (4.8) | 1,405 (5.8) | 1,470 (6.2) |
| Siblings | 0 | 7,628 (24.3) | 6,189 (22.7) | 3,581 (14.1) | 1,841 (7.6) | 1,355 (5.8) |
|  | 1 | 6,879 (21.9) | 6,454 (23.7) | 6,539 (25.7) | 5,838 (24.0) | 4,770 (20.2) |
|  | 2-3 | 10,246 (32.6) | 9,055 (33.2) | 9,281 (36.5) | 9,831 (40.4) | 10,192 (43.2) |
|  | 4-5 | 4,896 (15.6) | 4,097 (15.0) | 4,404 (17.3) | 4,939 (20.3) | 5,201 (22.1) |
|  | 6-12 | 1,743 (5.6) | 1,449 (5.3) | 1,642 (6.5) | 1,901 (7.8) | 2,056 (8.7) |
| **Paternal relatives** | | | | | | |
| Paternal aunt | 0 | 4,149 (25.3) | 3,706 (25.9) | 3,552 (26.6) | 3,509 (27.4) | 3,489 (28.1) |
|  | 1 | 4,439 (27.1) | 3,936 (27.5) | 3,689 (27.6) | 3,513 (27.4) | 3,406 (27.4) |
|  | 2-3 | 6,246 (38.1) | 5,365 (37.5) | 4,971 (37.2) | 4,720 (36.8) | 4,534 (36.5) |
|  | 4-9 | 1,573 (9.6) | 1,301 (9.1) | 1,168 (8.7) | 1,086 (8.5) | 998 (8.0) |
| Paternal uncle | 0 | 4,562 (27.8) | 4,079 (28.5) | 3,917 (29.3) | 3,853 (30.0) | 3,823 (30.8) |
|  | 1 | 4,691 (28.6) | 4,147 (29.0) | 3,905 (29.2) | 3,782 (29.5) | 3,675 (29.6) |
|  | 2-3 | 5,850 (35.7) | 5,000 (35.0) | 4,602 (34.4) | 4,314 (33.6) | 4,129 (33.2) |
|  | 4-9 | 1,304 (8.0) | 1,082 (7.6) | 956 (7.1) | 879 (6.9) | 800 (6.4) |
| Paternal cousins | 0 | 4,580 (27.9) | 3,791 (26.5) | 3,369 (25.2) | 3,044 (23.7) | 2,823 (22.7) |
|  | 1-5 | 6,198 (37.8) | 5,339 (37.3) | 4,963 (37.1) | 4,673 (36.4) | 4,443 (35.8) |
|  | 6-10 | 3,392 (20.7) | 3,039 (21.2) | 2,924 (21.9) | 2,963 (23.1) | 2,933 (23.6) |
|  | 11-15 | 1,490 (9.1) | 1,449 (10.1) | 1,419 (10.6) | 1,415 (11.0) | 1,466 (11.8) |
|  | 16-32 | 747 (4.6) | 690 (4.8) | 705 (5.3) | 733 (5.7) | 762 (6.1) |
| Paternal female cousins | 0 | 5,948 (36.3) | 4,970 (34.7) | 4,422 (33.1) | 4,019 (31.3) | 3,743 (30.1) |
|  | 1 | 2,450 (14.9) | 2,065 (14.4) | 1,935 (14.5) | 1,841 (14.4) | 1,760 (14.2) |
|  | 2-4 | 4,859 (29.6) | 4,369 (30.5) | 4,093 (30.6) | 4,015 (31.3) | 3,898 (31.4) |
|  | 5-7 | 2,091 (12.7) | 1,886 (13.2) | 1,910 (14.3) | 1,908 (14.9) | 1,952 (15.7) |
|  | 8+ | 1,059 (6.5) | 1,018 (7.1) | 1,020 (7.6) | 1,045 (8.2) | 1,074 (8.6) |
| Father’s sister’s daughter (pat. cousin) | 0 | 8,645 (52.7) | 7,353 (51.4) | 6,690 (50.0) | 6,237 (48.6) | 5,918 (47.6) |
|  | 1 | 2,643 (16.1) | 2,287 (16.0) | 2,118 (15.8) | 2,045 (15.9) | 1,953 (15.7) |
|  | 2-3 | 3,024 (18.4) | 2,743 (19.2) | 2,643 (19.8) | 2,619 (20.4) | 2,564 (20.6) |
|  | 4-9 | 2,095 (12.8) | 1,925 (13.5) | 1,929 (14.4) | 1,927 (15.0) | 1,992 (16.0) |
| Father’s brother’s daughter (pat. cousin) | 0 | 9,750 (59.4) | 8,259 (57.7) | 7,571 (56.6) | 7,061 (55.0) | 6,696 (53.9) |
|  | 1 | 2,320 (14.1) | 2,054 (14.4) | 1,919 (14.3) | 1,874 (14.6) | 1,827 (14.7) |
|  | 2-3 | 2,718 (16.6) | 2,490 (17.4) | 2,381 (17.8) | 2,370 (18.5) | 2,374 (19.1) |
|  | 4-9 | 1,619 (9.9) | 1,505 (10.5) | 1,509 (11.3) | 1,523 (11.9) | 1,530 (12.3) |
| Paternal male cousins | 0 | 5,852 (35.7) | 4,838 (33.8) | 4,343 (32.5) | 3,948 (30.8) | 3,666 (29.5) |
|  | 1 | 2,659 (16.2) | 2,241 (15.7) | 2,043 (15.3) | 1,961 (15.3) | 1,841 (14.8) |
|  | 2-4 | 4,803 (29.3) | 4,345 (30.4) | 4,155 (31.1) | 4,040 (31.5) | 3,994 (32.1) |
|  | 5-7 | 2,046 (12.5) | 1,909 (13.3) | 1,862 (13.9) | 1,876 (14.6) | 1,884 (15.2) |
|  | 8+ | 1,047 (6.4) | 975 (6.8) | 977 (7.3) | 1,003 (7.8) | 1,042 (8.4) |
| Father’s sister’s son (pat. cousin) | 0 | 8,558 (52.2) | 7,264 (50.8) | 6,644 (49.7) | 6,198 (48.3) | 5,858 (471.) |
|  | 1 | 2,529 (15.4) | 2,203 (15.4) | 2,039 (15.2) | 1,993 (15.5) | 1,950 (15.7) |
|  | 2-3 | 3,245 (19.8) | 2,928 (20.5) | 2,819 (21.1) | 2,717 (21.2) | 2,671 (21.5) |
|  | 4-9 | 2,075 (12.7) | 1,913 (13.4) | 1,878 (14.0) | 1,920 (15.0) | 1,948 (15.7) |
| Father’s brother’s son (pat. cousin) | 0 | 9,776 (59.6) | 8,249 (57.7) | 7,560 (56.5) | 7,042 (54.9) | 6,640 (53.4) |
|  | 1 | 2,391 (14.6) | 2,169 (15.2) | 2,017 (15.1) | 1,983 (15.5) | 1,953 (15.7) |
|  | 2-3 | 2,691 (16.4) | 2,447 (17.1) | 2,356 (17.6) | 2,319 (18.1) | 2,333 (18.8) |
|  | 4-9 | 1,549 (9.4) | 1,443 (10.1) | 1,447 (10.8) | 1,484 (11.6) | 1,501 (12.1) |
| Paternal grandmother | 0 | 7,817 (47.7) | 7,160 (50.1) | 6,989 (52.3) | 6,962 (54.4) | 7,028 (56.7) |
|  | 1 | 8,562 (52.3) | 7,121 (49.9) | 6,364 (47.7) | 5,839 (45.6) | 5,373 (43.3) |
| Paternal grandfather | 0 | 9,867 (61.1) | 8,932 (63.5) | 8,627 (65.6) | 8,521 (67.5) | 8,529 (69.8) |
|  | 1 | 6,271 (38.9) | 5,136 (36.5) | 4,531 (34.4) | 4,096 (32.5) | 3,695 (30.2) |
| Paternal great aunts | 0 | 5,280 (46.0) | 4,786 (47.5) | 4,594 (49.9) | 4,487 (50.0) | 4,470 (51.5) |
|  | 1 | 3,242 (28.2) | 2,813 (27.9) | 2,552 (27.2) | 2,441 (27.2) | 2,317 (26.7) |
|  | 2-7 | 2,958 (25.8) | 2,488 (24.7) | 2,236 (23.8) | 2,053 (22.9) | 1,901 (21.9) |
| Father’s mother’s sister (pat. great aunt) | 0 | 3,691 (51.0) | 3,335 (52.2) | 3,207 (53.8) | 3,108 (54.5) | 3,092 (56.0) |
|  | 1 | 1,961 (27.1) | 1,702 (26.7) | 1,548 (26.0) | 1,488 (26.1) | 1,415 (25.6) |
|  | 2-6 | 1,588 (21.9) | 1,349 (21.1) | 1,206 (20.2) | 1,105 (19.4) | 1,017 (18.4) |
| Father’s father’s sister (pat. great aunt) | 0 | 3,990 (56.2) | 3,606 (57.8) | 3,436 (59.2) | 3,362 (60.3) | 3,316 (61.5) |
|  | 1 | 1,766 (24.9) | 1,524 (24.4) | 1,374 (23.7) | 1,300 (23.3) | 1,221 (22.6) |
|  | 2-6 | 1,340 (18.9) | 1,107 (17.8) | 998 (17.2) | 9,15 (16.4) | 8,56 (15.9) |
| Paternal great uncles | 0 | 5,996 (52.2) | 5,421 (53.7) | 5,168 (55.1) | 5,078 (56.5) | 5,029 (57.9) |
|  | 1 | 3,217 (28.0) | 2,753 (27.3) | 2,543 (27.1) | 2,377 (26.5) | 2,247 (25.9) |
|  | 2-7 | 2,267 (19.8) | 1,913 (19.0) | 1,671 (17.8) | 1,526 (17.0) | 1,412 (16.3) |
| Father’s mother’s brother (pat. great uncle) | 0 | 4,099 (56.6) | 3,691 (57.8) | 3,501 (58.7) | 3,425 (60.1) | 3,376 (61.1) |
|  | 1 | 1,945 (26.9) | 1,697 (26.6) | 1,581 (26.5) | 1,489 (26.1) | 1,421 (25.7) |
|  | 2-7 | 1,196 (16.5) | 998 (15.6) | 879 (14.8) | 787 (13.8) | 727 (13.2) |
| Father’s father’s brother (pat. great uncle) | 0 | 4,377 (61.7) | 3,934 (63.1) | 3,763 (64.8) | 3,681 (66.0) | 3,628 (67.3) |
|  | 1 | 1,738 (24.5) | 1,479 (23.7) | 1,340 (23.1) | 1,248 (22.4) | 1,173 (21.8) |
|  | 2-6 | 9,81 (13.8) | 824 (13.2) | 705 (12.1) | 648 (11.6) | 592 (11.0) |
| Paternal great grandparents | 0 | 10,838 (94.4) | 9,595 (95.1) | 8,998 (95.9) | 8,670 (96.5) | 8,443 (97.2) |
|  | 1 | 642 (5.6) | 492 (4.9) | 384 (4.1) | 311 (3.5) | 245 (2.8) |
| Father’s mother’s mother (pat. great grandparent) | 0 | 6,954 (96.1) | 6,169 (96.6) | 5,789 (97.1) | 5,559 (97.5) | 5,418 (98.1) |
|  | 1 | 286 (3.9) | 217 (3.4) | 172 (2.9) | 142 (2.5) | 106 (1.9) |
| Father’s father’s mother (pat. great grandparent) | 0 | 6,881 (97.2) | 6,075 (97.6) | 5,683 (98.1) | 5,476 (98.4) | 5,311 (98.7) |
|  | 1 | 202 (2.8) | 149 (2.4) | 112 (1.93) | 88 (1.6) | 70 (1.3) |
| Father’s mother’s father (pat. great grandparent) | 0 | 7,049 (98.2) | 6,236 (98.5) | 5,839 (98.8) | 5,597 (99.0) | 5,438 (99.3) |
|  | 1 | 130 (1.8) | 95 (1.5) | 71 (1.2) | 57 (1.0) | 41 (0.7) |
| Father’s father’s father (pat. great grandparent) | 0 | 6,931 (98.3) | 6,100 (98.5) | 5,694 (98.7) | 5,480 (98.9) | 5,304 (99.0) |
|  | 1 | 119 (1.7) | 95 (1.5) | 76 (1.3) | 61 (1.1) | 54 (1.0) |
| **Maternal relatives** | | | | | | |
| Maternal aunt | 0 | 4,011 (23.8) | 3,581 (24.3) | 3,418 (24.8) | 3,310 (25.2) | 3,250 (25.6) |
|  | 1 | 4,611 (27.4) | 4,076 (27.7) | 3,829 (27.8) | 3,714 (28.3) | 3,630 (28.6) |
|  | 2-3 | 6,647 (39.5) | 5,769 (39.1) | 5,345 (38.8) | 5,042 (38.4) | 4,829 (38.0) |
|  | 4-8 | 1,575 (9.4) | 1,313 (8.9) | 1,169 (8.5) | 1,063 (8.1) | 994 (7.8) |
| Maternal uncle | 0 | 4,512 (26.8) | 4,042 (27.4) | 3,842 (27.9) | 3,719 (28.3) | 3,668 (28.9) |
|  | 1 | 4,652 (27.6) | 4,095 (27.8) | 3,865 (28.1) | 3,753 (28.6) | 36,55 (28.8) |
|  | 2-3 | 6,081 (36.1) | 5,248 (35.6) | 4,846 (35.2) | 4,551 (34.7) | 4,343 (34.2) |
|  | 4-9 | 1,599 (9.5) | 1,354 (9.2) | 1,208 (8.8) | 1,106 (8.4) | 1,036 (8.2) |
| Maternal cousins | 0 | 5,020 (29.8) | 4,153 (28.2) | 3,643 (26.5) | 3,235 (24.6) | 2,915 (23.0) |
|  | 1-5 | 6,322 (37.5) | 5,494 (37.3) | 5,095 (37.0) | 4,794 (36.5) | 4,580 (36.0) |
|  | 6-10 | 3,273 (19.4) | 3,007 (20.4) | 2,923 (21.2) | 2,955 (22.5) | 2,961 (23.3) |
|  | 11-15 | 1,509 (9.0) | 1,407 (9.6) | 1,408 (10.2) | 1,406 (10.7) | 1,476 (11.6) |
|  | 16-31 | 720 (4.3) | 678 (4.6) | 692 (5.0) | 739 (5.6) | 770 (6.1) |
| Maternal female cousins | 0 | 6,433 (38.2) | 5370 (36.4) | 4752 (34.5) | 4281 (32.6) | 3872 (30.5) |
|  | 1 | 2,539 (15.1) | 2,194 (14.9) | 2,004 (14.6) | 1,907 (14.5) | 1,859 (14.6) |
|  | 2-4 | 4,844 (28.8) | 4,322 (29.3) | 4,148 (30.1) | 4,036 (30.7) | 3,976 (31.3) |
|  | 5-7 | 2,087 (12.4) | 1,948 (13.2) | 1,952 (14.2) | 1,948 (14.8) | 1,985 (15.6) |
|  | 8+ | 941 (5.6) | 905 (6.1) | 905 (6.6) | 957 (7.3) | 1,010 (8.0) |
| Mother’s sister’s daughter (mat. cousin) | 0 | 9,371 (55.6) | 7,964 (54.0) | 7,217 (52.5) | 6,662 (50.7) | 6,245 (49.2) |
|  | 1 | 2,550 (15.1) | 2,250 (15.3) | 2,150 (15.6) | 2,051 (15.6) | 2,030 (16.0) |
|  | 2-3 | 3,012 (17.9) | 2,741 (18.6) | 2,654 (19.3) | 2,632 (20.0) | 2,572 (20.3) |
|  | 4-9 | 1,911 (11.4) | 1,784 (12.1) | 1,740 (12.6) | 1,784 (13.6) | 1,855 (14.6) |
| Mother’s brother’s daughter (mat. cousin) | 0 | 9,946 (59.1) | 8,550 (58.0) | 7,740 (56.3) | 7,184 (54.7) | 6,732 (53.0) |
|  | 1 | 2,402 (14.3) | 2,042 (13.9) | 1,957 (14.2) | 1,884 (14.4) | 1,839 (14.5) |
|  | 2-3 | 2,954 (17.5) | 2,674 (18.1) | 2,565 (18.6) | 2,510 (19.1) | 2,522 (19.9) |
|  | 4-9 | 1,542 (9.2) | 1,473 (10.0) | 1,499 (10.9) | 1,551 (11.8) | 1,609 (12.7) |
| Maternal male cousin | 0 | 6,346 (37.7) | 5,330 (36.2) | 4,709 (34.2) | 4,224 (32.2) | 3,868 (30.5) |
|  | 1 | 2,534 (15.0) | 2,168 (14.7) | 2,023 (14.7) | 1,872 (14.3) | 1,796 (14.1) |
|  | 2-4 | 4,848 (28.8) | 4,373 (29.7) | 4,153 (30.2) | 4,072 (31.0) | 4,012 (31.6) |
|  | 5-7 | 2,087 (12.4) | 1,910 (13.0) | 1,933 (14.1) | 1,968 (15.0) | 2,001 (15.8) |
|  | 8+ | 1,029 (6.1) | 958 (6.5) | 943 (6.9) | 993 (7.6) | 1,025 (8.1) |
| Mother’s sister’s son (mat. cousin) | 0 | 9,217 (54.7) | 7,898 (53.6) | 7,179 (52.2) | 6,628 (50.5) | 6,217 (49.0) |
|  | 1 | 2,585 (15.4) | 2,253 (15.3) | 2,132 (15.5) | 2,029 (15.5) | 2,001 (15.8) |
|  | 2-3 | 3,071 (18.2) | 2,783 (18.9) | 2,639 (19.2) | 2,624 (20.0) | 2,605 (20.5) |
|  | 4-9 | 1,971 (11.7) | 1,805 (12.3) | 1,811 (13.2) | 1,848 (14.1) | 2,879 (14.8) |
| Mother’s brother’s son (mat. cousin) | 0 | 9,878 (58.6) | 8,474 (57.5) | 7,652 (55.6) | 7,081 (53.9) | 6,633 (52.2) |
|  | 1 | 2,458 (14.6) | 2,156 (14.6) | 2,032 (14.8) | 1,965 (15.0) | 1,946 (15.3) |
|  | 2-3 | 2,897 (17.2) | 2,620 (17.8) | 2,574 (18.7) | 2,534 (19.3) | 2,533 (19.9) |
|  | 4-9 | 1,611 (9.6) | 1,489 (10.1) | 1,503 (10.9) | 1,549 (11.8) | 1,590 (12.5) |
| Maternal grandmother | 0 | 7,133 (42.4) | 6,485 (44.1) | 6,342 (46.2) | 6,309 (48.2) | 6,361 (50.2) |
|  | 1 | 9,679 (57.6) | 8,224 (55.9) | 7,390 (53.8) | 6,791 (51.8) | 6,315 (49.8) |
| Maternal grandfather | 0 | 9,387 (56.6) | 8,506 (58.6) | 8,262 (61.0) | 8,140 (63.0) | 8,133 (65.1) |
|  | 1 | 7,179 (43.4) | 6,004 (41.4) | 5,282 (39.9) | 4,780 (37.0) | 4,369 (34.9) |
| Maternal great aunts | 0 | 5,318 (43.1) | 4,753 (43.9) | 4,566 (45.3) | 4,474 (46.5) | 4,481 (48.2) |
|  | 1 | 3,461 (28.1) | 3,047 (28.2) | 2,819 (28.0) | 2,673 (27.8) | 2,534 (27.2) |
|  | 2-8 | 3,556 (28.8) | 3,021 (27.9) | 2,693 (26.7) | 2,467 (25.7) | 2,287 (24.6) |
| Mother’s mother’s sister (mat. great aunt) | 0 | 3,706 (49.2) | 3,327 (50.0) | 3,184 (51.3) | 3,096 (52.5) | 3,075 (53.9) |
|  | 1 | 1,934 (25.7) | 1,724 (25.9) | 1,585 (25.5) | 1,489 (25.2) | 1,408 (24.7) |
|  | 2-7 | 1,895 (25.2) | 1,607 (24.1) | 1,437 (23.2) | 1,316 (22.3) | 1,223 (21.4) |
| Mother’s father’s sister (mat. great aunt) | 0 | 4,015 (52.3) | 3,584 (53.2) | 3,432 (54.5) | 3,349 (55.6) | 3,328 (57.2) |
|  | 1 | 2,043 (26.6) | 1,772 (26.3) | 1,646 (26.2) | 1,555 (25.8) | 1,461 (25.1) |
|  | 2-7 | 1,621 (21.1) | 1,378 (20.5) | 1,217 (19.3) | 1,117 (18.6) | 1,032 (17.7) |
| Maternal great uncles | 0 | 6,056 (49.1) | 5,423 (50.1) | 5,180 (51.4) | 5,080 (52.8) | 5,043 (54.2) |
|  | 1 | 3,450 (28.0) | 3,015 (27.9) | 2,782 (27.6) | 2,599 (27.0) | 2,470 (26.6) |
|  | 2-8 | 2,829 (22.9) | 2,383 (22.0) | 2,116 (21.0) | 1,935 (20.1) | 1,789 (19.2) |
| Mother’s mother’s brother (mat. great uncle) | 0 | 4,081 (54.2) | 3,670 (55.1) | 3,499 (56.4) | 3,414 (57.9) | 3,367 (59.0) |
|  | 1 | 2,007 (26.6) | 1,759 (26.4) | 1,620 (26.1) | 1,503 (25.5) | 1,436 (25.2) |
|  | 2-7 | 1,447 (19.2) | 1,229 (18.5) | 1,087 (17.5) | 984 (16.7) | 903 (15.8) |
| Mother’s father’s brother (mat. great uncle) | 0 | 4,431 (57.7) | 3,963 (58.9) | 3,784 (60.1) | 3,687 (61.2) | 3,641 (62.6) |
|  | 1 | 1,930 (25.1) | 1,675 (24.9) | 1,536 (24.4) | 1,431 (23.8) | 1,341 (23.0) |
|  | 2-6 | 1,318 (17.2) | 1,096 (16.3) | 975 (15.5) | 903 (15.0) | 839 (14.4) |
| Maternal great grandparents | 0 | 11,372 (92.2) | 10,063 (93.0) | 9,470 (94.0) | 9,139 (95.0) | 8909 (95.8) |
|  | 1 | 963 (7.8) | 758 (7.0) | 608 (6.0) | 475 (5.0) | 393 (4.2) |
| Mother’s mother’s mother (mat. great grandparent) | 0 | 7,074 (93.9) | 6,286 (94.4) | 5,900 (95.1) | 5,664 (96.0) | 5,509 (96.6) |
|  | 1 | 461 (6.1) | 372 (5.6) | 306 (4.9) | 237 (4.0) | 197 (3.5) |
| Mother’s father’s mother (mat. great grandparent) | 0 | 7,346 (96.2) | 6,483 (96.8) | 6,092 (97.3) | 5,854 (97.8) | 5,678 (98.0) |
|  | 1 | 294 (3.9) | 215 (3.2) | 169 (2.7) | 134 (2.2) | 111 (1.9) |
| Mother’s mother’s father (mat. great grandparent) | 0 | 7,218 (97.2) | 6,401 (97.4) | 5,997 (97.9) | 5,726 (98.3) | 5,549 (98.6) |
|  | 1 | 212 (2.9) | 169 (2.6) | 128 (2.1) | 97 (1.7) | 80 (1.4) |
| Mother’s father’s father (mat. great grandparent) | 0 | 7,505 (98.2) | 6,602 (98.5) | 6,186 (98.7) | 5,935 (99.0) | 5,747 (99.2) |
|  | 1 | 141 (1.8) | 102 (1.5) | 83 (1.3) | 61 (1.0) | 49 (0.9) |

**Table S3.** Results from discrete time-event models of child mortality from birth to age of 5 years in relation to the number of each relative type present (alive). The presence of all relatives has been coded as a multilevel categorical variable, indicating the number of relatives of each type alive at the same time, in all other cases except in mother, father and grandparents where binary values have been used (0(dead)/1(alive)). P-values and X^2^_df_-values are given for the main variable (relative type). Other P-values are from pairwise comparisons with 0 relatives (of each specific type) as the reference category. Significant p-values (<0.05) are shown in bold.

| **Coding** | **Relative (alive)** | **n(obs)** | **n(children)** | **Estimate** | **OR (95% CLs)** | **X^2^ _df_** | **p-value** |
| --- | --- | --- | --- | --- | --- | --- | --- |
| 0/1 | Mother | 137,007 | 31,392 | -0.36 (-0.60, -0.11) | 0.70 (0.55, 0.90) | 6.05_1_ | **0.014** |
| 0/1 | Father | 137,007 | 31,392 | -0.19 (-0.40, 0.01) | 0.82 (0.67, 1.01) | 3.00_1_ | 0.083 |
| 0-8 | Brother | 137,007 | 31,392 |  |  | 3.59_3_ | 0.31 |
|  | 1 |  |  | 0.01 (-0.06, 0.07) | 1.01 (0.94, 1.08) |  | 0.81 |
|  | 2-3 |  |  | -0.05 (-0.13, 0.03) | 0.95 (0.88, 1.03) |  | 0.21 |
|  | 4-8 |  |  | -0.08 (-0.21, 0.05) | 0.93 (0.81, 1.05) |  | 0.24 |
| 0-9 | Sisters | 137,007 | 31,392 |  |  | 6.36_3_ | 0.10 |
|  | 1 |  |  | -0.08 (-0.15, -0.02) | 0.92 (0.86, 0.98) |  | **0.01** |
|  | 2-3 |  |  | -0.06 (-0.14, 0.01) | 0.94 (0.87, 1.01) |  | 0.11 |
|  | 4-9 |  |  | -0.08 (-0.21, 0.06) | 0.93 (0.81, 1.06) |  | 0.26 |
| 0-12 | Siblings | 137,007 | 31,392 |  |  | 6.84_4_ | 0.14 |
|  | 1 |  |  | -0.07 (-0.17, 0.03) | 0.93 (0.84, 1.03) |  | 0.16 |
|  | 2-3 |  |  | -0.08 (-0.19, 0.03) | 0.92 (0.83, 1.03) |  | 0.16 |
|  | 4-5 |  |  | -0.13 (-0.26, 0.01) | 0.88 (0.77, 1.01) |  | 0.07 |
|  | 6-12 |  |  | -0.21 (-0.37, -0.05) | 0.81 (0.69, 0.95) |  | **0.01** |
| Paternal relatives | | | | | | | |
| 0-9 | Paternal aunt | 69,350 | 16,407 |  |  | 9.64_3_ | **0.02** |
|  | 1 |  |  | 0.07 (-0.03, 0.17) | 1.07 (0.97, 1.19) |  | 0.16 |
|  | 2-3 |  |  | 0.13 (0.04, 0.22) | 1.14 (1.04, 1.25) |  | **0.006** |
|  | 4-9 |  |  | 0.17 (0.03, 0.31) | 1.18 (1.03, 1.36) |  | **0.02** |
| 0-9 | Paternal uncle | 69,350 | 16,407 |  |  | 14.12_3_ | **0.006** |
|  | 1 |  |  | 0.14 (0.04, 0.23) | 1.14 (1.04, 1.26) |  | **0.002** |
|  | 2-3 |  |  | 0.15 (0.06, 0.24) | 1.16 (1.06, 1.27) |  | **0.005** |
|  | 4-9 |  |  | 0.20 (0.06, 0.35) | 1.23 (1.06, 1.42) |  | **0.005** |
| 0-32 | Paternal cousins | 69,350 | 16,407 |  |  | 5.03_4_ | 0.28 |
|  | 1-5 |  |  | 0.10 (0.01, 0.19) | 1.10 (1.01, 1.21) |  | **0.03** |
|  | 6-10 |  |  | 0.05 (-0.06, 0.16) | 1.05 (0.94, 1.17) |  | 0.38 |
|  | 11-15 |  |  | 0.05 (-0.09, 0.18) | 1.05 (0.92, 1.2) |  | 0.47 |
|  | 16-32 |  |  | 0.08 (-0.09, 0.26) | 1.09 (0.92, 1.29) |  | 0.33 |
| 0-16 | Paternal female cousins | 69,350 | 16,407 |  |  | 8.35_4_ | 0.08 |
|  | 1 |  |  | 0.12 (0.02, 0.23) | 1.13 (1.02, 1.26) |  | **0.02** |
|  | 2-4 |  |  | 0.07 (-0.01, 0.16) | 1.08 (0.99, 1.17) |  | 0.10 |
|  | 5-7 |  |  | 0.00 (-0.12, 0.13) | 1.00 (0.89, 1.13) |  | 0.94 |
|  | 8+ |  |  | 0.13 (-0.01, 0.27) | 1.14 (0.99, 1.31) |  | 0.07 |
| 0-9 | Father’s sister’s daughter (pat. cousin) | 69,350 | 16,407 |  |  | 3.25_3_ | 0.36 |
|  | 1 |  |  | 0.03 (-0.06, 0.13) | 1.03 (0.94, 1.13) |  | 0.52 |
|  | 2-3 |  |  | -0.04 (-0.13, 0.05) | 0.96 (0.87, 1.06) |  | 0.41 |
|  | 4-9 |  |  | 0.06 (-0.05, 0.17) | 1.06 (0.95, 1.19) |  | 0.27 |
| 0-9 | Father’s brother’s daughter (pat. cousin) | 69,350 | 16,407 |  |  | 0.59_3_ | 0.90 |
|  | 1 |  |  | 0.02 (-0.08, 0.12) | 1.02 (0.92, 1.13) |  | 0.70 |
|  | 2-3 |  |  | 0.03 (-0.07, 0.12) | 1.03 (0.94, 1.13) |  | 0.56 |
|  | 4-9 |  |  | 0.04 (-0.08, 0.15) | 1.04 (0.92, 1.16) |  | 0.56 |
| 0-18 | Paternal male cousins | 69,350 | 16,407 |  |  | 1.81_4_ | 0.77 |
|  | 1 |  |  | 0.06 (-0.04, 0.17) | 1.06 (0.96, 1.18) |  | 0.25 |
|  | 2-4 |  |  | 0.01 (-0.09, 0.10) | 1.01 (0.92, 1.1) |  | 0.89 |
|  | 5-7 |  |  | 0.05 (-0.07, 0.17) | 1.05 (0.93, 1.18) |  | 0.42 |
|  | 8+ |  |  | 0.03 (-0.12, 0.17) | 1.03 (0.89, 1.19) |  | 0.71 |
| 0-9 | Father’s sister’s son (pat. cousin) | 69,350 | 16,407 |  |  | 0.82_3_ | 0.84 |
|  | 1 |  |  | 0.04 (-0.07, 0.14) | 1.04 (0.94, 1.15) |  | 0.49 |
|  | 2-3 |  |  | -0.00 (-0.10, 0.09) | 1.00 (0.91, 1.09) |  | 0.95 |
|  | 4-9 |  |  | 0.03 (-0.07, 0.14) | 1.03 (0.93, 1.15) |  | 0.54 |
| 0-9 | Father’s brother’s son (pat. cousin) | 69,350 | 16,407 |  |  | 1.73_3_ | 0.63 |
|  | 1 |  |  | 0.02 (-0.08, 0.11) | 1.02 (0.93, 1.12) |  | 0.71 |
|  | 2-3 |  |  | 0.02 (-0.07, 0.12) | 1.03 (0.93, 1.13) |  | 0.62 |
|  | 4-9 |  |  | -0.06 (-0.18, 0.06) | 0.94 (0.84, 1.06) |  | 0.34 |
| 0/1 | Paternal grandmother | 69,215 | 16,379 | 0.10 (0.03, 0.17) | 1.10 (1.03, 1.19) | 7.04_1_ | **0.008** |
| 0/1 | Paternal grandfather | 68,205 | 16,138 | 0.09 (0.01, 0.16) | 1.09 (1.01, 1.17) | 5.05_1_ | **0.02** |
| 0-7 | Paternal great aunts | 48,618 | 11,480 |  |  | 6.23_2_ | **0.04** |
|  | 1 |  |  | 0.12 (0.03, 0.22) | 1.13 (1.03, 1.25) |  | **0.01** |
|  | 2-7 |  |  | 0.03 (-0.08, 0.13) | 1.03 (0.93, 1.14) |  | 0.58 |
| 0-6 | Father’s mother’s sister (pat. great aunt) | 30,812 | 7,240 |  |  | 2.27_2_ | 0.32 |
|  | 1 |  |  | 0.09 (-0.04, 0.22) | 1.09 (0.96, 1.24) |  | 0.16 |
|  | 2-6 |  |  | -0.01 (-0.15, 0.13) | 0.99 (0.86, 1.14) |  | 0.89 |
| 0-6 | Father’s father’s sister (pat. great aunt) | 30,111 | 7,096 |  |  | 4.09_2_ | 0.13 |
|  | 1 |  |  | 0.12 (-0.00, 0.24) | 1.13 (1.00, 1.27) |  | 0.06 |
|  | 2-6 |  |  | 0.09 (-0.05, 0.23) | 1.10 (0.95, 1.26) |  | 0.20 |
| 0-7 | Paternal great uncles | 48,618 | 11,480 |  |  | 0.34_2_ | 0.84 |
|  | 1 |  |  | 0.03 (-0.07, 0.13) | 1.03 (0.93, 1.13) |  | 0.56 |
|  | 2-7 |  |  | 0.02 (-0.10, 0.13) | 1.02 (0.9, 1.14) |  | 0.79 |
| 0-7 | Father’s mother’s brother (pat. great uncle) | 30,812 | 7,240 |  |  | 4.22_2_ | 0.12 |
|  | 1 |  |  | -0.11 (-0.24, 0.02) | 0.90 (0.79, 1.02) |  | 0.09 |
|  | 2-7 |  |  | 0.05 (-0.10, 0.21) | 1.05 (0.90, 1.23) |  | 0.52 |
| 0-6 | Father’s father’s brother (pat. great uncle) | 30,111 | 7,096 |  |  | 5.84_2_ | **0.05** |
|  | 1 |  |  | 0.16 (0.03, 0.28) | 1.17 (1.03, 1.32) |  | **0.01** |
|  | 2-6 |  |  | 0.02 (-0.14, 0.18) | 1.02 (0.87, 1.20) |  | 0.81 |
| *Maternal relatives* | | | | | | | |
| 0-8 | Maternal aunt | 71,175 | 16,844 |  |  | 1.35_3_ | 0.72 |
|  | 1 |  |  | 0.01 (-0.09, 0.11) | 1.01 (0.91, 1.12) |  | 0.82 |
|  | 2-3 |  |  | 0.05 (-0.05, 0.14) | 1.05 (0.95, 1.15) |  | 0.33 |
|  | 4-8 |  |  | 0.06 (-0.09, 0.20) | 1.06 (0.91, 1.22) |  | 0.45 |
| 0-9 | Maternal uncle | 71,175 | 16,844 |  |  | 4.49_3_ | 0.21 |
|  | 1 |  |  | 0.05 (-0.05, 0.15) | 1.05 (0.95, 1.16) |  | 0.31 |
|  | 2-3 |  |  | 0.09 (-0.01, 0.18) | 1.09 (0.99, 1.20) |  | 0.07 |
|  | 4-9 |  |  | 0.12 (-0.01, 0.25) | 1.13 (0.99, 1.29) |  | 0.08 |
| 0-31 | Maternal cousins | 71,175 | 16,844 |  |  | 7.10_4_ | 0.13 |
|  | 1-5 |  |  | 0.08 (-0.01, 0.17) | 1.08 (0.99, 1.18) |  | 0.09 |
|  | 6-10 |  |  | 0.14 (0.03, 0.25) | 1.15 (1.03, 1.28) |  | **0.01** |
|  | 11-15 |  |  | 0.12 (-0.02, 0.25) | 1.12 (0.98, 1.29) |  | 0.09 |
|  | 16-31 |  |  | 0.15 (-0.03, 0.33) | 1.17 (0.97, 1.40) |  | 0.09 |
| 0-17 | Maternal female cousins | 71,175 | 16,844 |  |  | 5.02_4_ | 0.29 |
|  | 1 |  |  | 0.08 (-0.03, 0.18) | 1.08 (0.97, 1.20) |  | 0.16 |
|  | 2-4 |  |  | 0.09 (0.00, 0.18) | 1.10 (1.00, 1.20) |  | **0.05** |
|  | 5-7 |  |  | 0.10 (-0.01, 0.21) | 1.10 (0.99, 1.24) |  | 0.09 |
|  | 8+ |  |  | 0.06 (-0.10, 0.23) | 1.07 (0.90, 1.26) |  | 0.44 |
| 0-9 | Mother’s sister’s daughter (mat. cousin) | 71,175 | 16,844 |  |  | 2.23_3_ | 0.53 |
|  | 1 |  |  | 0.05 (-0.05, 0.15) | 1.05 (0.95, 1.17) |  | 0.34 |
|  | 2-3 |  |  | 0.06 (-0.04, 0.15) | 1.06 (0.97, 1.16) |  | 0.23 |
|  | 4-9 |  |  | 0.06 (-0.05, 0.18) | 1.06 (0.95, 1.19) |  | 0.29 |
| 0-9 | Mother’s brother’s daughter (mat. cousin) | 71,175 | 16,844 |  |  | 5.91_3_ | 0.12 |
|  | 1 |  |  | 0.10 (-0.00, 0.19) | 1.10 (1.00, 1.22) |  | 0.06 |
|  | 2-3 |  |  | 0.10 (0.00, 0.19) | 1.10 (1.00, 1.21) |  | **0.047** |
|  | 4-9 |  |  | 0.07 (-0.05, 0.19) | 1.07 (0.95, 1.21) |  | 0.24 |
| 0-18 | Maternal male cousin | 71,175 | 16,844 |  |  | 14.67_4_ | **0.005** |
|  | 1 |  |  | 0.03 (-0.08, 0.14) | 1.03 (0.93, 1.15) |  | 0.57 |
|  | 2-4 |  |  | 0.12 (0.03, 0.21) | 1.13 (1.03, 1.24) |  | **0.007** |
|  | 5-7 |  |  | 0.03 (-0.09, 0.15) | 1.03 (0.92, 1.17) |  | 0.58 |
|  | 8+ |  |  | 0.24 (0.09, 0.38) | 1.27 (1.10, 1.46) |  | **0.001** |
| 0-9 | Mother’s sister’s son (mat. cousin) | 71,175 | 16,844 |  |  | 2.23_3_ | 0.53 |
|  | 1 |  |  | 0.02 (-0.08, 0.13) | 1.02 (0.92, 1.13) |  | 0.67 |
|  | 2-3 |  |  | 0.07 (-0.03, 0.16) | 1.07 (0.97, 1.18) |  | 0.17 |
|  | 4-9 |  |  | 0.06 (-0.05, 0.17) | 1.06 (0.95, 1.19) |  | 0.30 |
| 0-9 | Mother’s brother’s son (mat. cousin) | 71,175 | 16,844 |  |  | 5.72_3_ | 0.13 |
|  | 1 |  |  | 0.02 (-0.08, 0.12) | 1.02 (0.92, 1.12) |  | 0.73 |
|  | 2-3 |  |  | 0.07 (-0.02, 0.16) | 1.07 (0.98, 1.17) |  | 0.15 |
|  | 4-9 |  |  | 0.14 (0.02, 0.26) | 1.15 (1.02, 1.30) |  | **0.02** |
| 0/1 | Maternal grandmother | 71,029 | 16,812 | -0.06 (-0.14, 0.01) | 0.94 (0.87, 1.01) | 3.02_1_ | 0.08 |
| 0/1 | Maternal grandfather | 70,060 | 16,584 | 0.05 (-0.03, 0.12) | 1.05 (0.97, 1.13) | 1.42_1_ | 0.23 |
| 0-8 | Maternal great aunts | 52,150 | 12,335 |  |  | 2.24_2_ | 0.33 |
|  | 1 |  |  | -0.05 (-0.15, 0.04) | 0.95 (0.86, 1.05) |  | 0.28 |
|  | 2-8 |  |  | 0.03 (-0.07, 0.13) | 1.03 (0.93, 1.14) |  | 0.58 |
| 0-7 | Mother’s mother’s sister (mat. great aunt) | 32,006 | 7,535 |  |  | 0.38_2_ | 0.83 |
|  | 1 |  |  | 0.04 (-0.09, 0.17) | 1.04 (0.92, 1.18) |  | 0.54 |
|  | 2-7 |  |  | 0.02 (-0.12, 0.15) | 1.02 (0.88, 1.16) |  | 0.87 |
| 0-7 | Mother’s father’s sister (mat. great aunt) | 32,550 | 7,679 |  |  | 2.18_2_ | 0.34 |
|  | 1 |  |  | -0.04 (-0.17, 0.08) | 0.96 (0.84, 1.09) |  | 0.51 |
|  | 2-7 |  |  | 0.08 (-0.07, 0.22) | 1.08 (0.94, 1.25) |  | 0.28 |
| 0-8 | Maternal great uncles | 52,150 | 12,335 |  |  | 0.64_2_ | 0.73 |
|  | 1 |  |  | 0.04 (-0.06, 0.13) | 1.04 (0.95, 1.14) |  | 0.44 |
|  | 2-8 |  |  | 0.02 (-0.09, 0.14) | 1.03 (0.92, 1.15) |  | 0.67 |
| 0-7 | Mother’s mother’s brother (mat. great uncle) | 32,006 | 7,535 |  |  | 0.42_2_ | 0.81 |
|  | 1 |  |  | 0.01 (-0.11, 0.13) | 1.01 (0.89, 1.13) |  | 0.90 |
|  | 2-7 |  |  | 0.05 (-0.10, 0.20) | 1.05 (0.90, 1.22) |  | 0.52 |
| 0-6 | Mother’s father’s brother (mat. great uncle) | 32,550 | 7,679 |  |  | 3.67_2_ | 0.16 |
|  | 1 |  |  | 0.12 (-0.00, 0.24) | 1.13 (1.00, 1.27) |  | **0.05** |
|  | 2-6 |  |  | 0.03 (-0.14, 0.19) | 1.03 (0.87, 1.21) |  | 0.76 |

**Table S4.** Results from discrete time-event models of child mortality from birth to age of 5 years in relation to the presence of each relative type. The presence of all relatives has been coded as 0/1, i.e. none vs. at least one specific type of relative alive. Significant P-values (<0.05) are shown in bold. int.=interaction with the relative variable. age=child age, SES=family socioeconomic status.

| **Coding** | **Relative (alive)** | **n(obs)** | **n(children)** | **Estimate** | **OR (95% CLs)** | **X^2^ _df_** | **p-value** | **age-int.** | **SES-int.** |  |
| --- | --- | --- | --- | --- | --- | --- | --- | --- | --- | --- |
| 0/1 | Mother | 137,007 | 31,392 | -0.36 (-0.60, -0.11) | 0.70 (0.55, 0.90) | 6.05_1_ | **0.014** | **0.017** | 0.32 |  |
| 0/1 | Father | 137,007 | 31,392 | -0.19 (-0.40, 0.01) | 0.82 (0.67, 1.01) | 3.00_1_ | 0.083 | 0.59 | 0.22 |  |
| 0/1 | Brothers | 137,007 | 31,392 | -0.01 (-0.08, 0.05) | 0.99 (0.93, 1.05) | 0.18_1_ | 0.67 | 0.55 | 0.07 |  |
| 0/1 | Sisters | 137,007 | 31,392 | -0.08 (-0.14, -0.01) | 0.93 (0.87, 0.99) | 5.82_1_ | **0.016** | 0.62 | 0.23 |  |
| 0/1 | Siblings | 137,007 | 31,392 | -0.08 (-0.17, 0.02) | 0.93 (0.84, 1.02) | 2.27_1_ | 0.13 | **0.006** | 0.08 |  |
| *Paternal relatives* | | | | | | | | | | |
| 0/1 | Paternal aunt | 69,350 | 16,407 | 0.11 (0.03, 0.20) | 1.12 (1.03, 1.22) | 7.22_1_ | **0.007** | 0.32 | 0.35 |  |
| 0/1 | Paternal uncle | 69,350 | 16,407 | 0.15 (0.07, 0.23) | 1.16 (1.07, 1.26) | 13.24_1_ | **0.0003** | 0.07 | 0.17 |  |
| 0/1 | Paternal cousin | 69,350 | 16,407 | 0.08 (-0.01, 0.16) | 1.08 (0.99, 1.18) | 3.34_1_ | 0.07 | 0.17 | **0.03** |  |
| 0/1 | Paternal female cousin | 69,350 | 16,407 | 0.08 (0.00, 0.15) | 1.08 (1.00, 1.17) | 3.97_1_ | **0.047** | 0.39 | **0.03** |  |
| 0/1 | Father’s sister’s daughter (pat. cousin) | 69,350 | 16,407 | 0.01 (-0.06, 0.09) | 1.01 (0.94, 1.09) | 0.12_1_ | 0.73 | 0.62 | 0.13 |  |
| 0/1 | Father’s brother’s daughter (pat. cousin) | 69,350 | 16,407 | 0.03 (-0.04, 0.10) | 1.03 (0.96, 1.10) | 0.55_1_ | 0.46 | 0.31 | 0.54 |  |
| 0/1 | Paternal male cousin | 69,350 | 16,407 | 0.03 (-0.05, 0.11) | 1.03 (0.95, 1.12) | 0.56_1_ | 0.45 | 0.17 | 0.20 |  |
| 0/1 | Father’s sister’s son (pat. cousin) | 69,350 | 16,407 | 0.02 (-0.05, 0.09) | 1.02 (0.95, 1.10) | 0.27_1_ | 0.60 | 0.70 | 0.27 |  |
| 0/1 | Father’s brother’s son (pat. cousin) | 69,350 | 16,407 | 0.00 (-0.07, 0.08) | 1.00 (0.93, 1.08) | 0.01_1_ | 0.92 | 0.28 | **0.04** |  |
| 0/1 | Paternal grandmother | 69,215 | 16,379 | 0.10 (0.03, 0.17) | 1.10 (1.03, 1.19) | 7.04_1_ | **0.008** | 0.07 | 0.32 |  |
| 0/1 | Paternal grandfather | 68,205 | 16,138 | 0.09 (0.01, 0.16) | 1.09 (1.01, 1.17) | 5.05_1_ | **0.02** | 0.14 | 0.98 |  |
| 0/1 | Paternal great aunts | 48,618 | 11,480 | 0.08 (-0.00, 0.17) | 1.09 (0.99, 1.18) | 3.71_1_ | 0.05 | 0.24 | 0.93 |  |
| 0/1 | Father’s mother’s sister (pat. great aunt) | 30,812 | 7,240 | 0.05 (-0.06, 0.16) | 1.05 (0.94, 1.17) | 0.77_1_ | 0.38 | 0.90 | 0.89 |  |
| 0/1 | Father’s father’s sister (pat. great aunt) | 30,111 | 7,096 | 0.11 (0.00, 0.21) | 1.11 (1.00, 1.24) | 3.95_1_ | **0.047** | 0.06 | 0.78 |  |
| 0/1 | Paternal great uncles | 48,618 | 11,480 | 0.02 (-0.06, 0.11) | 1.02 (0.94, 1.11) | 0.30_1_ | 0.58 | **0.03** | 0.13 |  |
| 0/1 | Father’s mother’s brother (pat. great uncle) | 30,812 | 7,240 | -0.05 (-0.16, 0.06) | 0.95 (0.85, 1.06) | 0.77_1_ | 0.38 | 0.17 | 0.42 |  |
| 0/1 | Father’s father’s brother (pat. great uncle) | 30,111 | 7,096 | 0.11 (0.00, 0.22) | 1.12 (1.00, 1.25) | 4.05_1_ | **0.04** | 0.52 | 0.19 |  |
| *Maternal relatives* | | | | | | | | | | |
| 0/1 | Maternal aunt | 71,175 | 16,844 | 0.04 (-0.05, 0.12) | 1.04 (0.95, 1.13) | 0.73_1_ | 0.39 | 0.37 | 0.12 |  |
| 0/1 | Maternal uncle | 71,175 | 16,844 | 0.08 (-0.01, 0.16) | 1.08 (0.99, 1.18) | 3.28_1_ | 0.07 | 0.11 | 0.28 |  |
| 0/1 | Maternal cousin | 71,175 | 16,844 | 0.10 (0.02, 0.19) | 1.11 (1.02, 1.21) | 5.68_1_ | **0.02** | 0.09 | 0.40 |  |
| 0/1 | Maternal female cousin | 71,175 | 16,844 | 0.09 (0.01, 0.17) | 1.09 (1.01, 1.18) | 4.74_1_ | **0.03** | 0.19 | 0.45 |  |
| 0/1 | Mother’s sister’s daughter (mat. cousin) | 71,175 | 16,844 | 0.06 (-0.02, 0.13) | 1.06 (0.98, 1.14) | 2.21_1_ | 0.14 | 0.15 | 0.55 |  |
| 0/1 | Mother’s brother’s daughter (mat. cousin) | 71,175 | 16,844 | 0.09 (0.02, 0.17) | 1.10 (1.02, 1.18) | 5.71_1_ | **0.02** | **0.03** | 0.68 |  |
| 0/1 | Maternal male cousin | 71,175 | 16,844 | 0.09 (0.01, 0.17) | 1.10 (1.01, 1.19) | 5.50_1_ | **0.02** | 0.14 | 0.49 |  |
| 0/1 | Mother’s sister’s son (mat. cousin) | 71,175 | 16,844 | 0.05 (-0.02, 0.12) | 1.05 (0.98, 1.13) | 1.71_1_ | 0.19 | 0.05 | 0.57 |  |
| 0/1 | Mother’s brother’s son (mat. cousin) | 71,175 | 16,844 | 0.07 (-0.01, 0.14) | 1.07 (0.99, 1.15) | 3.19_1_ | 0.07 | 0.08 | 0.99 |  |
| 0/1 | Maternal grandmother | 71,029 | 16,812 | -0.06 (-0.14, 0.01) | 0.94 (0.87, 1.01) | 3.02_1_ | 0.08 | **0.03** | **0.009** |  |
| 0/1 | Maternal grandfather | 70,060 | 16,584 | 0.05 (-0.03, 0.12) | 1.05 (0.97, 1.13) | 1.42_1_ | 0.23 | 0.69 | 0.32 |  |
| 0/1 | Maternal great aunts | 52,150 | 12,335 | -0.01 (-0.10, 0.07) | 0.99 (0.91, 1.07) | 0.10_1_ | 0.75 | 0.69 | 0.53 |  |
| 0/1 | Mother’s mother’s sister (mat. great aunt) | 32,006 | 7,535 | 0.03 (-0.08, 0.13) | 1.03 (0.92, 1.14) | 0.23_1_ | 0.63 | 0.88 | 0.47 |  |
| 0/1 | Mother’s father’s sister (mat. great aunt) | 32,550 | 7,679 | 0.01 (-0.10, 0.12) | 1.01 (0.91, 1.12) | 0.02_1_ | 0.88 | 0.27 | 0.59 |  |
| 0/1 | Maternal great uncles | 52,150 | 12,335 | 0.03 (-0.05, 0.11) | 1.03 (0.95, 1.12) | 0.57_1_ | 0.45 | 0.23 | 0.50 |  |
| 0/1 | Mother’s mother’s brother (mat. great uncle) | 32,006 | 7,535 | 0.02 (-0.08, 0.13) | 1.02 (0.92, 1.14) | 0.20_1_ | 0.66 | 0.77 | 0.21 |  |
| 0/1 | Mother’s father’s brother (mat. great uncle) | 32,550 | 7,679 | 0.08 (-0.03, 0.19) | 1.09 (0.98, 1.21) | 2.26_1_ | 0.13 | 0.29 | 0.41 |  |

**Table S5.** Results from discrete-time event models of child mortality from birth to age of 5 years, which adjust for missing-% of each relative type presence, in relation to the number of each relative type present. The presence of all relatives has been coded as a multilevel categorical variable, indicating the number of relatives of each type alive at the same time, in all other cases except in mother, father and grandparents where binary values have been used (0(dead)/1(alive)). P-values and X^2^_df_-values are given for the main variable (relative type). Other P-values are from pairwise comparisons with 0 relatives (of each specific type) as the reference category. Significant p-values (<0.05) are shown in bold.

| **Coding** | **Relative (alive)** | **n(obs)** | **n(children)** | **Estimate** | **OR (95% CLs)** | **X^2^ _df_** | **p-value** |
| --- | --- | --- | --- | --- | --- | --- | --- |
| 0/1 | Mother | 137,007 | 31,392 | -0.45 (-0.71, -0.19) | 0.64 (0.49, 0.82) | 8.34_1_ | **0.004** |
| 0/1 | Father | 137,007 | 31,392 | -0.19 (-0.40, 0.02) | 0.83 (0.67, 1.02) | 2.60_1_ | 0.11 |
| 0-8 | Brother | 137,007 | 31,392 |  |  | 3.55_1_ | 0.31 |
|  | 1 |  |  | 0.01 (-0.06, 0.07) | 1.01 (0.94, 1.07) |  | 0.80 |
|  | 2-3 |  |  | -0.05 (-0.13, 0.03) | 0.95 (0.88, 1.03) |  | 0.22 |
|  | 4-8 |  |  | -0.08 (-0.20, 0.05) | 0.92 (0.82, 1.05) |  | 0.24 |
| 0-9 | Sisters | 137,007 | 31,392 |  |  | 6.23_3_ | 0.10 |
|  | 1 |  |  | -0.08 (-0.15, -0.02) | 0.92 (0.86, 0.98) |  | **0.01** |
|  | 2-3 |  |  | -0.06 (-0.14, 0.02) | 0.94 (0.87, 1.02) |  | 0.12 |
|  | 4-9 |  |  | -0.07 (-0.20, 0.06) | 0.93 (0.82, 1.06) |  | 0.27 |
| 0-12 | Siblings | 137,007 | 31,392 |  |  | 6.43_4_ | 0.17 |
|  | 1 |  |  | -0.07 (-0.17, 0.03) | 0.93 (0.84, 1.03) |  | 0.18 |
|  | 2-3 |  |  | -0.07 (-0.18, 0.04) | 0.93 (0.84, 1.04) |  | 0.19 |
|  | 4-5 |  |  | -0.12 (-0.26, 0.02) | 0.89 (0.77, 1.02) |  | 0.09 |
|  | 6-12 |  |  | -0.20 (-0.36, -0.04) | 0.82 (0.70, 0.96) |  | **0.02** |
| *Paternal relatives* | | | | | | | |
| 0-9 | Paternal aunt | 69,350 | 16,407 |  |  | 9.22_3_ | **0.03** |
|  | 1 |  |  | 0.07 (-0.03, 0.17) | 1.07 (0.97, 1.19) |  | 0.16 |
|  | 2-3 |  |  | 0.13 (0.03, 0.22) | 1.14 (1.03, 1.25) |  | **0.008** |
|  | 4-9 |  |  | 0.17 (0.03, 0.31) | 1.19 (1.03, 1.36) |  | **0.02** |
| 0-9 | Paternal uncle | 69,350 | 16,407 |  |  | 13.50_3_ | **0.004** |
|  | 1 |  |  | 0.13 (0.04, 0.23) | 1.14 (1.04, 1.26) |  | **0.006** |
|  | 2-3 |  |  | 0.15 (0.05, 0.24) | 1.16 (1.05, 1.27) |  | **0.002** |
|  | 4-9 |  |  | 0.20 (0.06, 0.35) | 1.22 (1.06, 1.42) |  | **0.006** |
| 0-32 | Paternal cousins | 69,350 | 16,407 |  |  | 5.83_4_ | 0.21 |
|  | 1-5 |  |  | 0.11 (0.02, 0.20) | 1.12 (1.02, 1.22) |  | 0.02 |
|  | 6-10 |  |  | 0.06 (-0.05, 0.17) | 1.06 (0.95, 1.19) |  | 0.29 |
|  | 11-15 |  |  | 0.06 (-0.08, 0.19) | 1.06 (0.92, 1.21) |  | 0.40 |
|  | 16-32 |  |  | 0.09 (-0.08, 0.26) | 1.09 (0.92, 1.30) |  | 0.29 |
| 0-16 | Paternal female cousins | 69,350 | 16,407 |  |  | 8.68_4_ | 0.07 |
|  | 1 |  |  | 0.13 (0.02, 0.23) | 1.14 (1.02, 1.26) |  | **0.02** |
|  | 2-4 |  |  | 0.08 (-0.01, 0.16) | 1.08 (0.99, 1.17) |  | 0.09 |
|  | 5-7 |  |  | 0.01 (-0.11, 0.13) | 1.01 (0.90, 1.14) |  | 0.86 |
|  | 8+ |  |  | 0.13 (-0.01, 0.27) | 1.14 (0.99, 1.31) |  | 0.06 |
| 0-9 | Father’s sister’s daughter (pat. cousin) | 69,350 | 16,407 |  |  | 3.28_3_ | 0.35 |
|  | 1 |  |  | 0.03 (-0.06, 0.13) | 1.03 (0.94, 1.14) |  | 0.47 |
|  | 2-3 |  |  | -0.04 (-0.13, 0.06) | 0.96 (0.88, 1.06) |  | 0.41 |
|  | 4-9 |  |  | 0.06 (-0.05, 0.17) | 1.06 (0.95, 1.19) |  | 0.28 |
| 0-9 | Father’s brother’s daughter (pat. cousin) | 69,350 | 16,407 |  |  | 0.58_3_ | 0.90 |
|  | 1 |  |  | 0.02 (-0.08, 0.12) | 1.02 (0.92, 1.13) |  | 0.68 |
|  | 2-3 |  |  | 0.03 (-0.07, 0.12) | 1.03 (0.93, 1.13) |  | 0.57 |
|  | 4-9 |  |  | 0.03 (-0.08, 0.15) | 1.03 (0.92, 1.16) |  | 0.56 |
| 0-18 | Paternal male cousins | 69,350 | 16,407 |  |  | 1.85_4_ | 0.76 |
|  | 1 |  |  | 0.06 (-0.04, 0.17) | 1.06 (0.96, 1.19) |  | 0.24 |
|  | 2-4 |  |  | 0.01 (-0.08, 0.10) | 1.01 (0.92, 1.11) |  | 0.84 |
|  | 5-7 |  |  | 0.05 (-0.07, 0.17) | 1.05 (0.93, 1.19) |  | 0.41 |
|  | 8+ |  |  | 0.03 (-0.12, 0.18) | 1.03 (0.89, 1.20) |  | 0.67 |
| 0-9 | Father’s sister’s son (pat. cousin) | 69,350 | 16,407 |  |  | 0.80_3_ | 0.85 |
|  | 1 |  |  | 0.04 (-0.07, 0.14) | 1.04 (0.93, 1.15) |  | 0.49 |
|  | 2-3 |  |  | -0.00 (-0.10, 0.09) | 1.00 (0.90, 1.09) |  | 0.93 |
|  | 4-9 |  |  | 0.03 (-0.08, 0.14) | 1.03 (0.92, 1.15) |  | 0.56 |
| 0-9 | Father’s brother’s son (pat. cousin) | 69,350 | 16,407 |  |  | 1.76_3_ | 0.62 |
|  | 1 |  |  | 0.02 (-0.08, 0.12) | 1.02 (0.92, 1.13) |  | 0.69 |
|  | 2-3 |  |  | 0.03 (-0.07, 0.12) | 1.03 (0.93, 1.13) |  | 0.62 |
|  | 4-9 |  |  | -0.06 (-0.18, 0.06) | 0.94 (0.84, 1.06) |  | 0.34 |
| 0/1 | Paternal grandmother | 69,215 | 16,379 | 0.05 (-0.02, 0.13) | 1.05 (0.98, 1.14) | 2.14_1_ | 0.14 |
| 0/1 | Paternal grandfather | 68,205 | 16,138 | 0.06 (-0.01, 0.14) | 1.06 (0.99, 1.15) | 2.67_1_ | 0.10 |
| 0-7 | Paternal great aunts | 48,618 | 11,480 |  |  | 5.87_2_ | **0.05** |
|  | 1 |  |  | 0.12 (0.02, 0.22) | 1.13 (1.02, 1.25) |  | **0.02** |
|  | 2-7 |  |  | 0.02 (-0.08, 0.13) | 1.02 (0.92, 1.14) |  | 0.65 |
| 0-6 | Father’s mother’s sister (pat. great aunt) | 30,812 | 7,240 |  |  | 2.18_2_ | 0.34 |
|  | 1 |  |  | 0.09 (-0.04, 0.21) | 1.09 (0.96, 1.23) |  | 0.18 |
|  | 2-6 |  |  | -0.01 (-0.15, 0.13) | 0.99 (0.86, 1.14) |  | 0.86 |
| 0-6 | Father’s father’s sister (pat. great aunt) | 30,111 | 7,096 |  |  | 4.28_2_ | 0.12 |
|  | 1 |  |  | 0.12 (-0.00, 0.24) | 1.13 (1.00, 1.27) |  | **0.05** |
|  | 2-6 |  |  | 0.09 (-0.05, 0.24) | 1.09 (0.95, 1.27) |  | 0.19 |
| 0-7 | Paternal great uncles | 48,618 | 11,480 |  |  | 0.38_2_ | 0.83 |
|  | 1 |  |  | 0.03 (-0.07, 0.13) | 1.03 (0.93, 1.14) |  | 0.55 |
|  | 2-7 |  |  | 0.02 (-0.10, 0.13) | 1.02 (0.90, 1.14) |  | 0.77 |
| 0-7 | Father’s mother’s brother (pat. great uncle) | 30,812 | 7,240 |  |  | 4.42_2_ | 0.11 |
|  | 1 |  |  | -0.11 (-0.23, 0.02) | 0.90 (0.79, 1.02) |  | 0.09 |
|  | 2-7 |  |  | 0.06 (-0.10, 0.21) | 1.06 (0.90, 1.23) |  | 0.46 |
| 0-6 | Father’s father’s brother (pat. great uncle) | 30,111 | 7,096 |  |  | 5.78_2_ | 0.06 |
|  | 1 |  |  | 0.16 (0.03, 0.28) | 1.17 (1.03, 1.32) |  | **0.01** |
|  | 2-6 |  |  | 0.02 (-0.14, 0.18) | 1.02 (0.87, 1.20) |  | 0.83 |
| *Maternal relatives* | | | | | | | |
| 0-8 | Maternal aunt | 71,175 | 16,844 |  |  | 1.04_3_ | 0.79 |
|  | 1 |  |  | 0.01 (-0.09, 0.11) | 1.01 (0.91, 1.12) |  | 0.89 |
|  | 2-3 |  |  | 0.04 (-0.05, 0.13) | 1.04 (0.95, 1.14) |  | 0.40 |
|  | 4-8 |  |  | 0.05 (-0.10, 0.19) | 1.05 (0.90, 1.21) |  | 0.54 |
| 0-9 | Maternal uncle | 71,175 | 16,844 |  |  | 3.35_3_ | 0.34 |
|  | 1 |  |  | 0.05 (-0.05, 0.15) | 1.05 (0.95, 1.16) |  | 0.34 |
|  | 2-3 |  |  | 0.08 (-0.02, 0.17) | 1.08 (0.98, 1.19) |  | 0.12 |
|  | 4-9 |  |  | 0.10 (-0.03, 0.24) | 1.11 (0.97, 1.27) |  | 0.12 |
| 0-31 | Maternal cousins | 71,175 | 16,844 |  |  | 7.36_4_ | 0.12 |
|  | 1-5 |  |  | 0.08 (-0.01, 0.17) | 1.08 (0.99, 1.19) |  | 0.08 |
|  | 6-10 |  |  | 0.14 (0.03, 0.25) | 1.15 (1.03, 1.28) |  | **0.01** |
|  | 11-15 |  |  | 0.12 (-0.02, 0.26) | 1.13 (0.98, 1.30) |  | 0.08 |
|  | 16-31 |  |  | 0.16 (-0.02, 0.34) | 1.17 (0.98, 1.40) |  | 0.09 |
| 0-17 | Maternal female cousins | 71,175 | 16,844 |  |  | 5.34_4_ | 0.25 |
|  | 1 |  |  | 0.08 (-0.03, 0.19) | 1.08 (0.97, 1.21) |  | 0.15 |
|  | 2-4 |  |  | 0.09 (0.00, 0.18) | 1.09 (1.00, 1.20) |  | **0.04** |
|  | 5-7 |  |  | 0.10 (-0.01, 0.22) | 1.11 (0.99, 1.25) |  | 0.07 |
|  | 8+ |  |  | 0.07 (-0.09, 0.24) | 1.07 (0.91, 1.27) |  | 0.37 |
| 0-9 | Mother’s sister’s daughter (mat. cousin) | 71,175 | 16,844 |  |  | 2.20_3_ | 0.53 |
|  | 1 |  |  | 0.05 (-0.05, 0.15) | 1.05 (0.95, 1.16) |  | 0.34 |
|  | 2-3 |  |  | 0.06 (-0.04, 0.15) | 1.06 (0.96, 1.16) |  | 0.23 |
|  | 4-9 |  |  | 0.06 (-0.05, 0.18) | 1.06 (0.95, 1.20) |  | 0.29 |
| 0-9 | Mother’s brother’s daughter (mat. cousin) | 71,175 | 16,844 |  |  | 6.10_3_ | 0.11 |
|  | 1 |  |  | 0.10 (-0.00, 0.20) | 1.11 (1.00, 1.22) |  | **0.05** |
|  | 2-3 |  |  | 0.10 (0.00, 0.19) | 1.11 (1.00, 1.21) |  | **0.04** |
|  | 4-9 |  |  | 0.07 (-0.05, 0.19) | 1.07 (0.95, 1.21) |  | 0.24 |
| 0-18 | Maternal male cousin | 71,175 | 16,844 |  |  | 14.58_4_ | **0.005** |
|  | 1 |  |  | 0.03 (-0.08, 0.14) | 1.03 (0.92, 1.15) |  | 0.58 |
|  | 2-4 |  |  | 0.12 (0.03, 0.21) | 1.13 (1.03, 1.23) |  | **0.008** |
|  | 5-7 |  |  | 0.03 (-0.09, 0.15) | 1.03 (0.91, 1.16) |  | 0.59 |
|  | 8+ |  |  | 0.23 (0.09, 0.38) | 1.26 (1.09, 1.46) |  | **0.001** |
| 0-9 | Mother’s sister’s son (mat. cousin) | 71,175 | 16,844 |  |  | 2.25_3_ | 0.52 |
|  | 1 |  |  | 0.02 (-0.08, 0.12) | 1.02 (0.92, 1.13) |  | 0.67 |
|  | 2-3 |  |  | 0.07 (-0.03, 0.16) | 1.07 (0.97, 1.17) |  | 0.17 |
|  | 4-9 |  |  | 0.06 (-0.05, 0.17) | 1.06 (0.95, 1.19) |  | 0.30 |
| 0-9 | Mother’s brother’s son (mat. cousin) | 71,175 | 16,844 |  |  | 5.69_3_ | 0.13 |
|  | 1 |  |  | 0.02 (-0.08, 0.12) | 1.02 (0.92, 1.13) |  | 0.71 |
|  | 2-3 |  |  | 0.07 (-0.02, 0.16) | 1.07 (0.98, 1.17) |  | 0.15 |
|  | 4-9 |  |  | 0.14 (0.02, 0.26) | 1.15 (1.02, 1.30) |  | **0.02** |
| 0/1 | Maternal grandmother | 71,029 | 16,812 | -0.08 (-0.16, -0.01) | 0.92 (0.85, 0.99) | 4.70_1_ | **0.03** |
| 0/1 | Maternal grandfather | 70,060 | 16,584 | 0.02 (-0.06, 0.09) | 1.02 (0.94, 1.09) | 0.17_1_ | 0.68 |
| 0-8 | Maternal great aunts | 52,150 | 12,335 |  |  | 2.05_2_ | 0.36 |
|  | 1 |  |  | -0.06 (-0.16, 0.04) | 0.94 (0.85, 1.04) |  | 0.25 |
|  | 2-8 |  |  | 0.02 (-0.08, 0.12) | 1.02 (0.92, 1.13) |  | 0.73 |
| 0-7 | Mother’s mother’s sister (mat. great aunt) | 32,006 | 7,535 |  |  | 0.38_2_ | 0.83 |
|  | 1 |  |  | 0.04 (-0.09, 0.17) | 1.04 (0.91, 1.19) |  | 0.53 |
|  | 2-7 |  |  | 0.01 (-0.12, 0.15) | 1.02 (0.89, 1.16) |  | 0.86 |
| 0-7 | Mother’s father’s sister (mat. great aunt) | 32,550 | 7,679 |  |  | 1.73_2_ | 0.42 |
|  | 1 |  |  | -0.05 (-0.18, 0.08) | 0.95 (0.84, 1.08) |  | 0.44 |
|  | 2-7 |  |  | 0.06 (-0.09, 0.20) | 1.06 (0.91, 1.22) |  | 0.43 |
| 0-8 | Maternal great uncles | 52,150 | 12,335 |  |  | 0.65_2_ | 0.72 |
|  | 1 |  |  | 0.04 (-0.06, 0.13) | 1.04 (0.94, 1.14) |  | 0.43 |
|  | 2-8 |  |  | 0.03 (-0.09, 0.14) | 1.03 (0.91, 1.15) |  | 0.66 |
| 0-7 | Mother’s mother’s brother (mat. great uncle) | 32,006 | 7,535 |  |  | 0.46_2_ | 0.79 |
|  | 1 |  |  | 0.01 (-0.11, 0.13) | 1.01 (0.90, 1.14) |  | 0.90 |
|  | 2-7 |  |  | 0.05 (-0.10, 0.20) | 1.05 (0.90, 1.22) |  | 0.49 |
| 0-6 | Mother’s father’s brother (mat. great uncle) | 32,550 | 7,679 |  |  | 3.60_2_ | 0.17 |
|  | 1 |  |  | 0.12 (-0.00, 0.24) | 1.13 (1.00, 1.27) |  | 0.06 |
|  | 2-6 |  |  | 0.02 (-0.14, 0.19) | 1.02 (0.87, 1.21) |  | 0.79 |

**Table S6.** Results from discrete-time event models of child mortality from birth to age of 5 years, which adjust for missing-% of each relative type presence, in relation to the presence of each relative type. The presence of all relatives has been coded as 0/1, i.e. none vs. at least one specific type of relative alive. Significant P-values (<0.05) are shown in bold. int.=interaction with the relative variable. age=child age, SES=family socioeconomic status.

| **Coding** | **Relative (alive)** | **n(obs)** | **n(children)** | **Estimate** | **OR (95% CLs)** | **X^2^ _df_** | **p-value** | **age-int.** | **SES-int.** |  |
| --- | --- | --- | --- | --- | --- | --- | --- | --- | --- | --- |
| 0/1 | Mother | 137,007 | 31,392 | -0.45 (-0.71, -0.19) | 0.64 (0.49, 0.82) | 8.34_1_ | **0.004** | **0.009** | 0.43 |  |
| 0/1 | Father | 137,007 | 31,392 | -0.19 (-0.40, 0.02) | 0.83 (0.67, 1.02) | 3.00_1_ | 0.11 | 0.53 | 0.19 |  |
| 0/1 | Brothers | 137,007 | 31,392 | -0.01 (-0.08, 0.05) | 0.99 (0.92, 1.05) | 0.16_1_ | 0.69 | 0.55 | 0.07 |  |
| 0/1 | Sisters | 137,007 | 31,392 | -0.07 (-0.14, -0.01) | 0.93 (0.87, 0.99) | 5.66_1_ | **0.017** | 0.62 | 0.22 |  |
| 0/1 | Siblings | 137,007 | 31,392 | -0.07 (-0.17, 0.03) | 0.93 (0.84, 1.03) | 1.98_1_ | 0.16 | **0.005** | 0.07 |  |
| *Paternal relatives* | | | | | | | | | | |
| 0/1 | Paternal aunt | 69,350 | 16,407 | 0.11 (0.03, 0.20) | 1.12 (1.03, 1.22) | 6.85_1_ | **0.009** | 0.32 | 0.35 |  |
| 0/1 | Paternal uncle | 69,350 | 16,407 | 0.15 (0.06, 0.23) | 1.16 (1.06, 1.26) | 12.65_1_ | **0.0004** | 0.07 | 0.16 |  |
| 0/1 | Paternal cousin | 69,350 | 16,407 | 0.08 (-0.01, 0.16) | 1.08 (0.99, 1.17) | 3.44_1_ | 0.06 | 0.17 | **0.03** |  |
| 0/1 | Paternal female cousin | 69,350 | 16,407 | 0.08 (0.01, 0.16) | 1.08 (1.02, 1.17) | 4.44_1_ | **0.04** | 0.38 | **0.03** |  |
| 0/1 | Father’s sister’s daughter (pat. cousin) | 69,350 | 16,407 | 0.01 (-0.06, 0.09) | 1.01 (0.94, 1.09) | 0.14_1_ | 0.71 | 0.62 | 0.13 |  |
| 0/1 | Father’s brother’s daughter (pat. cousin) | 69,350 | 16,407 | 0.03 (-0.04, 0.10) | 1.03 (0.96, 1.11) | 0.55_1_ | 0.46 | 0.31 | 0.52 |  |
| 0/1 | Paternal male cousin | 69,350 | 16,407 | 0.03 (-0.05, 0.11) | 1.03 (0.96, 1.12) | 0.66_1_ | 0.41 | 0.17 | 0.19 |  |
| 0/1 | Father’s sister’s son (pat. cousin) | 69,350 | 16,407 | 0.02 (-0.05, 0.09) | 1.02 (0.95, 1.09) | 0.24_1_ | 0.62 | 0.70 | 0.26 |  |
| 0/1 | Father’s brother’s son (pat. cousin) | 69,350 | 16,407 | 0.00 (-0.07, 0.08) | 1.00 (0.93, 1.08) | 0.01_1_ | 0.91 | 0.28 | **0.04** |  |
| 0/1 | Paternal grandmother | 69,215 | 16,379 | 0.05 (-0.02, 0.13) | 1.05 (0.98, 1.14) | 2.14_1_ | 0.14 | 0.07 | 0.39 |  |
| 0/1 | Paternal grandfather | 68,205 | 16,138 | 0.06 (-0.01, 0.14) | 1.06 (0.99, 1.15) | 2.67_1_ | 0.10 | 0.14 | 0.95 |  |
| 0/1 | Paternal great aunts | 48,618 | 11,480 | 0.08 (-0.01, 0.16) | 1.08 (0.99, 1.17) | 3.30_1_ | 0.07 | 0.24 | 0.92 |  |
| 0/1 | Father’s mother’s sister (pat. great aunt) | 30,812 | 7,240 | 0.05 (-0.06, 0.15) | 1.05 (0.94, 1.16) | 0.68_1_ | 0.41 | 0.90 | 0.88 |  |
| 0/1 | Father’s father’s sister (pat. great aunt) | 30,111 | 7,096 | 0.11 (0.00, 0.22) | 1.12 (1.00, 1.25) | 4.14_1_ | **0.04** | 0.06 | 0.80 |  |
| 0/1 | Paternal great uncles | 48,618 | 11,480 | 0.03 (-0.06, 0.11) | 1.03 (0.94, 1.12) | 0.34_1_ | 0.56 | **0.03** | 0.14 |  |
| 0/1 | Father’s mother’s brother (pat. great uncle) | 30,812 | 7,240 | -0.05 (-0.16, 0.06) | 0.95 (0.85, 1.06) | 0.71_1_ | 0.40 | 0.17 | 0.44 |  |
| 0/1 | Father’s father’s brother (pat. great uncle) | 30,111 | 7,096 | 0.11 (0.00, 0.22) | 1.12 (1.00, 1.25) | 3.97_1_ | **0.05** | 0.52 | 0.19 |  |
| *Maternal relatives* | | | | | | | | | | |
| 0/1 | Maternal aunt | 71,175 | 16,844 | 0.03 (-0.06, 0.11) | 1.03 (0.94, 1.12) | 0.44_1_ | 0.51 | 0.37 | 0.12 |  |
| 0/1 | Maternal uncle | 71,175 | 16,844 | 0.07 (-0.02, 0.15) | 1.07 (0.98, 1.16) | 2.51_1_ | 0.11 | 0.11 | 0.22 |  |
| 0/1 | Maternal cousin | 71,175 | 16,844 | 0.10 (0.02, 0.19) | 1.11, 1.02, 1.16) | 5.49_1_ | **0.02** | 0.09 | 0.38 |  |
| 0/1 | Maternal female cousin | 71,175 | 16,844 | 0.09 (0.01, 0.17) | 1.09 (1.01, 1.19) | 5.11_1_ | **0.02** | 0.19 | 0.46 |  |
| 0/1 | Mother’s sister’s daughter (mat. cousin) | 71,175 | 16,844 | 0.06 (-0.02, 0.13) | 1.06 (0.98, 1.14) | 2.18_1_ | 0.14 | 0.15 | 0.55 |  |
| 0/1 | Mother’s brother’s daughter (mat. cousin) | 71,175 | 16,844 | 0.09 (0.02, 0.17) | 1.09 (1.02, 1.19) | 5.86_1_ | **0.02** | **0.03** | 0.65 |  |
| 0/1 | Maternal male cousin | 71,175 | 16,844 | 0.09 (0.01, 0.17) | 1.09 (1.01, 1,19) | 5.37_1_ | **0.02** | 0.14 | 0.49 |  |
| 0/1 | Mother’s sister’s son (mat. cousin) | 71,175 | 16,844 | 0.05 (-0.02, 0.12) | 1.05 (0.98, 1.13) | 1.72_1_ | 0.19 | **0.05** | 0.57 |  |
| 0/1 | Mother’s brother’s son (mat. cousin) | 71,175 | 16,844 | 0.07 (-0.01, 0.14) | 1.07 (0.99, 1.15) | 3.23_1_ | 0.07 | 0.08 | 0.99 |  |
| 0/1 | Maternal grandmother | 71,029 | 16,812 | -0.08 (-0.16, -0.01) | 0.92 (0.85, 0.99) | 4.70_1_ | **0.03** | **0.03** | **0.01** |  |
| 0/1 | Maternal grandfather | 70,060 | 16,584 | 0.02 (-0.06, 0.09) | 1.02 (0.94, 1.09) | 0.17_1_ | 0.68 | 0.69 | 0.45 |  |
| 0/1 | Maternal great aunts | 52,150 | 12,335 | -0.02 (-0.10, 0.06) | 0.98 (0.90, 1.06) | 0.25_1_ | 0.62 | 0.68 | 0.55 |  |
| 0/1 | Mother’s mother’s sister (mat. great aunt) | 32,006 | 7,535 | 0.03 (-0.08, 0.13) | 1.03 (0.92, 1.14) | 0.24_1_ | 0.62 | 0.87 | 0.46 |  |
| 0/1 | Mother’s father’s sister (mat. great aunt) | 32,550 | 7,679 | 0.01 (-0.10, 0.12) | 1.01 (0.90, 1.13) | 0.01_1_ | 0.94 | 0.27 | 0.66 |  |
| 0/1 | Maternal great uncles | 52,150 | 12,335 | 0.03 (-0.05, 0.12) | 1.03 (0.95, 1.13) | 0.58_1_ | 0.45 | 0.23 | 0.50 |  |
| 0/1 | Mother’s mother’s brother (mat. great uncle) | 32,006 | 7,535 | 0.03 (-0.08, 0.13) | 1.03 (0.92, 1.14) | 0.22_1_ | 0.64 | 0.77 | 0.20 |  |
| 0/1 | Mother’s father’s brother (mat. great uncle) | 32,550 | 7,679 | 0.08 (-0.03, 0.19) | 1.08 (0.97, 1.21) | 2.15_1_ | 0.14 | 0.29 | 0.41 |  |

**Figure S1.** Presence of different relative types from birth to age 5. A) Presence of same generation relatives with child age and B) presence of older generation relatives with child age.

a


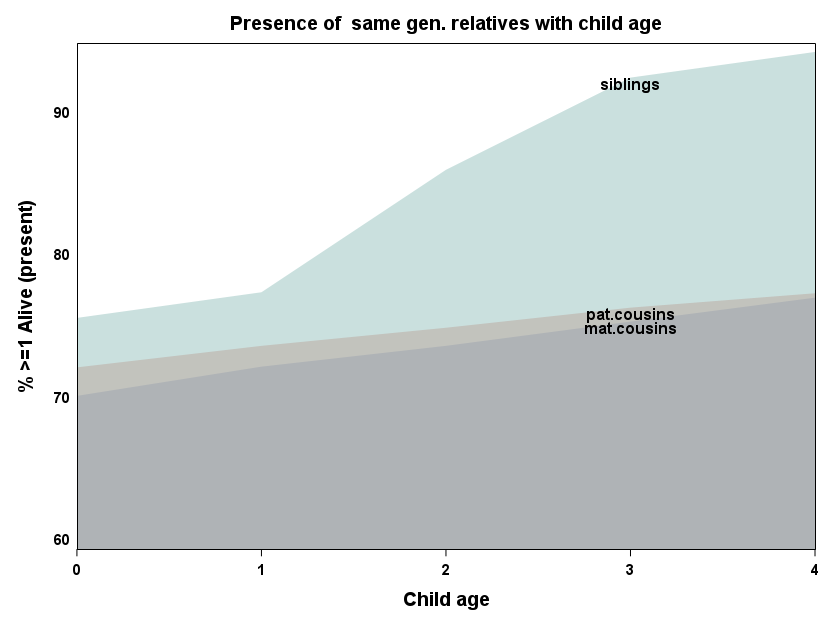


b


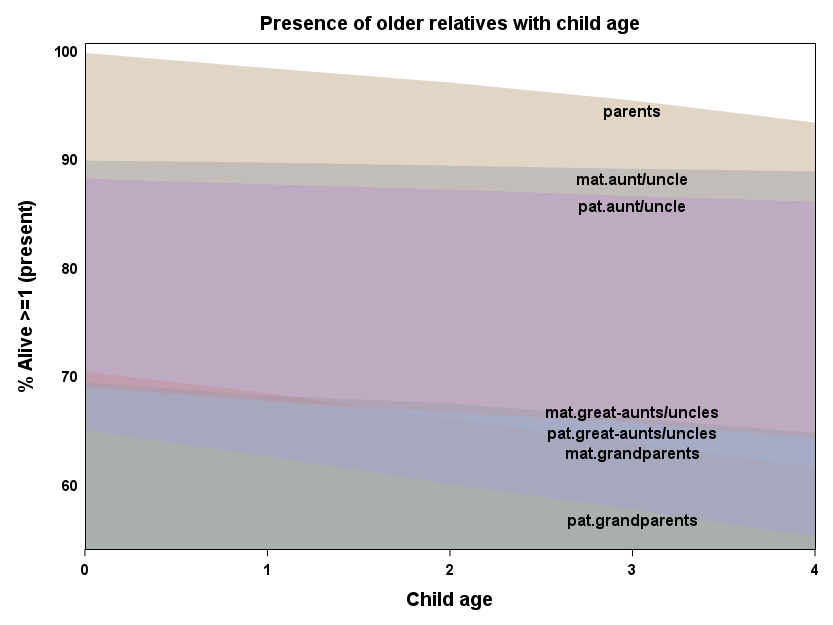


**Figure S2**. Presence of some specific types of relatives was differently associated with child mortality depending on the child’s age (child age-interaction).


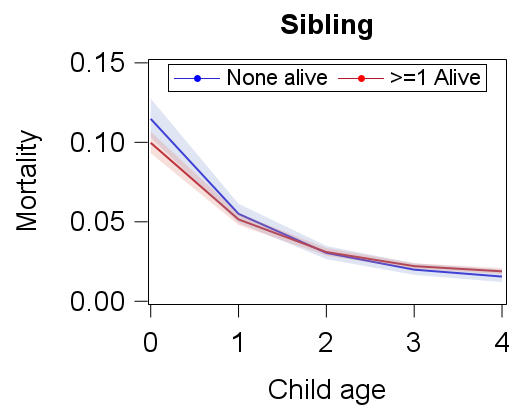

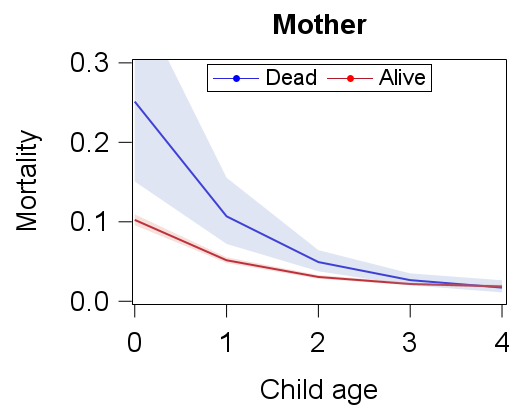
a b


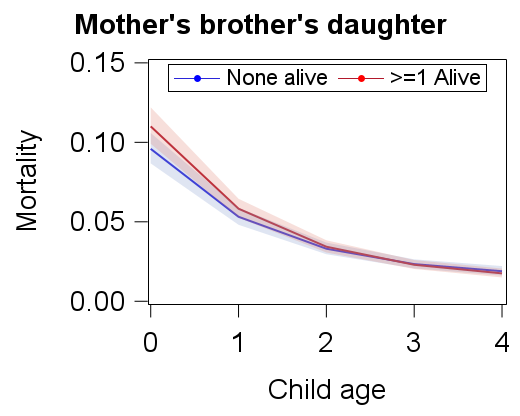

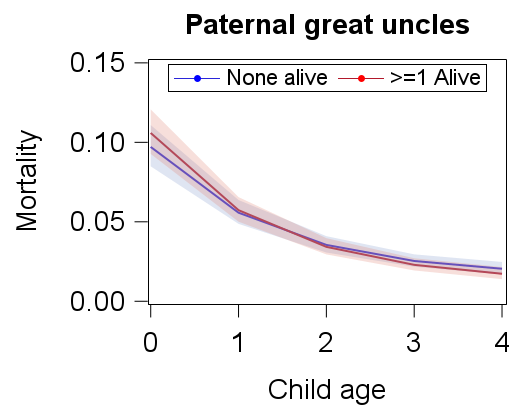
c d


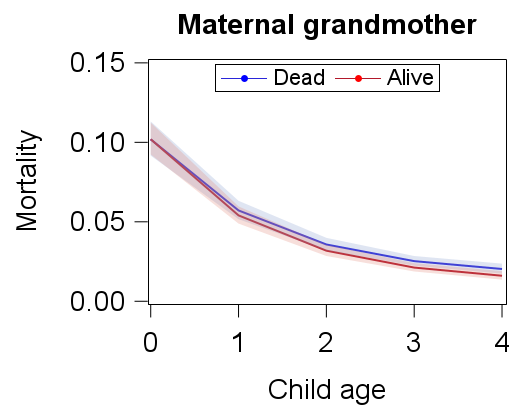
e

**Figure S3.** Presence of some specific types of relatives was differently associated with child mortality depending on the family SES (family socioeconomic status-interaction).


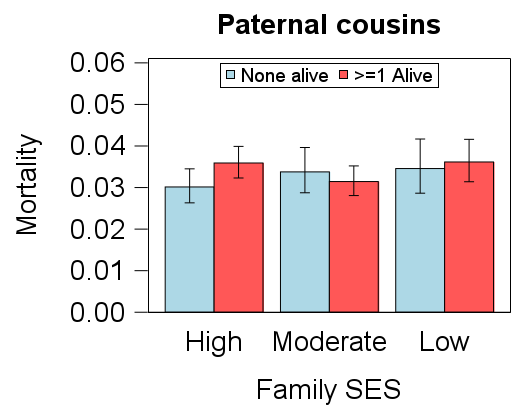

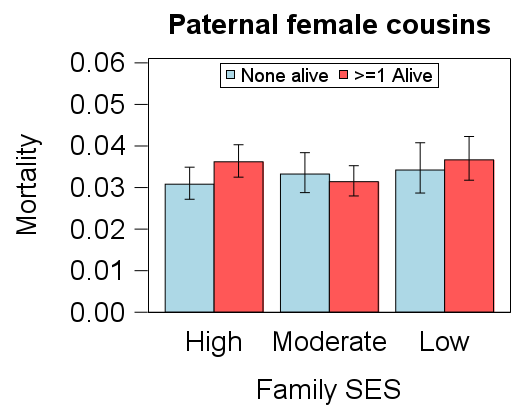
a b

|  | OR (95% CLs) | p-value |
| --- | --- | --- |
| High | 1.18 (1.06, 1.31) | **0.002** |
| Moderate | 0.94 (0.83, 1.08) | 0.39 |
| Low | 1.07 (0.89, 1.29) | 0.45 |

|  | OR (95% CLs) | p-value |
| --- | --- | --- |
| High | 1.20 (1.07, 1.35) | **0.002** |
| Moderate | 0.93 (0.80, 1.08) | 0.33 |
| Low | 1.05 (0.86, 1.27) | 0.64 |


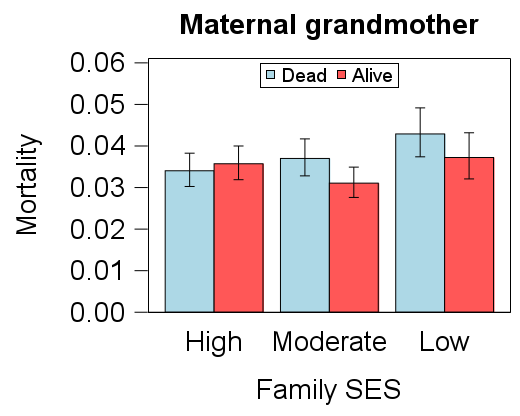

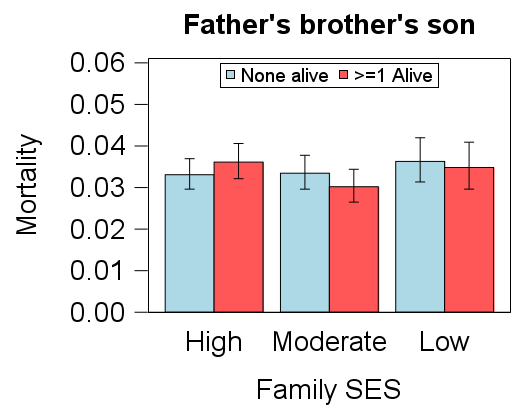
c d

|  | OR (95% CLs) | p-value |
| --- | --- | --- |
| High | 1.10 (0.99, 1.21) | 0.07 |
| Moderate | 0.90 (0.79, 1.02) | 0.10 |
| Low | 0.96 (0.81, 1.13) | 0.62 |

|  | OR (95% CLs) | p-value |
| --- | --- | --- |
| High | 1.05 (0.95, 1.17) | 0.35 |
| Moderate | 0.83 (0.74, 0.94) | **0.003** |
| Low | 0.86 (0.74, 1.01) | 0.07 |
